# Accelerated discovery of high-performance Al-Si-Mg-Sc casting alloys by integrating active learning with high-throughput CALPHAD calculations

**DOI:** 10.1080/14686996.2023.2196242

**Published:** 2023-04-11

**Authors:** Jianbao Gao, Jing Zhong, Guangchen Liu, Shaoji Zhang, Jiali Zhang, Zuming Liu, Bo Song, Lijun Zhang

**Affiliations:** a State Key Laboratory of Powder Metallurgy, Central South University, Changsha, Hunan, P.R. China; b Mechanical and Materials Engineering Department, Worcester Polytechnic Institute, Worcester, MA, USA; c State Key Laboratory of Materials Processing and Die & Mould Technology, School of Materials Science and Engineering, Huazhong University of Science and Technology, WH, P.R. China

**Keywords:** Alloy design, casting aluminum alloy, high-throughput calculations, CALPHAD, active learning

## Abstract

Scandium is the best alloying element to improve the mechanical properties of industrial Al-Si-Mg casting alloys. Most literature reports devote to exploring/designing optimal Sc additions in different commercial Al-Si-Mg casting alloys with well-defined compositions. However, no attempt to optimize the contents of Si, Mg, and Sc has been made due to the great challenge of simultaneous screening in high-dimensional composition space with limited experimental data. In this paper, a novel alloy design strategy was proposed and successfully applied to accelerate the discovery of hypoeutectic Al-Si-Mg-Sc casting alloys over high-dimensional composition space. Firstly, high-throughput CALculation of PHAse Diagrams (CALPHAD) solidification simulations of ocean of hypoeutectic Al-Si-Mg-Sc casting alloys over a wide composition range were performed to establish the quantitative relation ‘composition-process-microstructure’. Secondly, the relation ‘microstructure-mechanical properties’ of Al-Si-Mg-Sc hypoeutectic casting alloys was acquired using the active learning technique supported by key experiments designed by CALPHAD and Bayesian optimization samplings. After a benchmark in A356-xSc alloys, such a strategy was utilized to design the high-performance hypoeutectic Al-xSi-yMg alloys with optimal Sc additions that were later experimentally validated. Finally, the present strategy was successfully extended to screen the optimal contents of Si, Mg, and Sc over high-dimensional hypoeutectic Al-xSi-yMg-zSc composition space. It is anticipated that the proposed strategy integrating active learning with high-throughput CALPHAD simulations and key experiments should be generally applicable to the efficient design of high-performance multi-component materials over high-dimensional composition space.

## GRAPHICAL ABSTRACT

**Figure:**
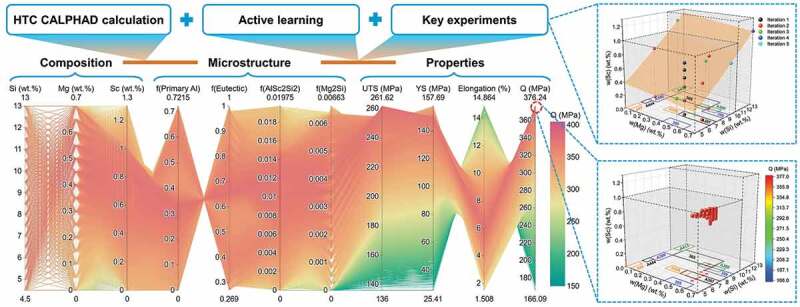

## 1. Introduction

With the high strength-to-weight ratio, excellent castability, and excellent thermal/electrical conductivities, Al-Si-Mg casting alloys are widely used in the automotive industries, aerospace, construction, and electricity industries [1]. Continuous improvement in the properties of Al-Si-Mg casting alloy is required to meet the increasing consumer demand for high-performance devices. The mechanical properties of casting light alloys are determined by the solidification microstructures, including the morphology, grain size, grain size distribution, and volume fraction of the primary phase, eutectic phases, and precipitates. However, for traditional Al-Si-Mg casting alloys, their microstructures typically consist of coarse anisotropic dendritic (Al) phase, need-like eutectic (Si), and brittle intermetallics (e.g. β-Al_5_FeSi), will greatly limit the mechanical properties of casting alloys [2]. The addition of alloying/microalloying elements for regulating the cast microstructure has been serving as one effective method to improve the comprehensive properties of casting alloy. In recent decades, rare earth elements (Sc [3–16], La [17], Ce [18], Er [19], Y [20], etc.) were widely introduced to greatly modify the eutectic (Si) from coarse need-like to fine-fibrous and refine α-(Al) to significantly improve the mechanical properties and castability of Al-Si-Mg-based casting alloys. Especially, the rare earth element Sc might be the most effective modifier for eutectic (Si) [21], which could be due to the decreased surface tension of molten Al [22,23] or the competitive nucleation and growth among the reactive components in ternary or higher-order eutectics [11,15,24]. Moreover, when the ratio of *w*(Si)/*w*(Sc) is lower than 2.5 (i.e. Al-2Si-1Sc alloy [25]), one can observe the primary Al_3_Sc, which is coherent with the (Al) at low lattice misfit [26] and has a high resistance to coarsen [27] to refine the (Al) grains and AlSc_2_Si_2_ phase via the peritectic reaction (L+Al_3_Sc→AlSi_2_Sc_2_) [28]. While the primary AlSc_2_Si_2_ may form at 2.5 < *w*(Si)/*w*(Sc) < 4.5 [28] (see Supplementary Tables S1, S2, and Figure S1). When the ratio of *w*(Si)/*w*(Sc) is larger than 4.5, one can see the formation of eutectic AlSc_2_Si_2_, which is partially coherent with primary/eutectic (Al) [15] and can enhance the mechanical properties due to its large Bulk modulus (B = 99.9 GPa) and shear modulus (G = 74.1 GPa) [29].

Accordingly, numerous studies have been devoted to optimizing the additional content of Sc in different casting aluminum alloys with well-defined compositions by means of the experimental ‘trial-and-error’ method. For example, Muhammad et al. [30] and Pramod et al. [31] studied the effect of different Sc additions (0.0, 0.2, 0.4 wt.% Sc) on microstructure and mechanical properties of A357 and A356 casting alloys, respectively, and the experimental results showed that the mechanical properties were improved with the increase of Sc addition under the experimental maximum addition (0.4 wt.%). Pandee et al. [13] also studied the effect of different Sc additions (0, 0.24, 0.40, 0.65 wt.% Sc) on the microstructure and mechanical properties of A356 (Al-7Si-0.3 Mg) casting alloy, and the results showed that 0.4 wt.% Sc was the optimal addition for A356 casting alloy. Whereas the results from Xu et al. [32] showed that the optimal Sc addition was 0.8 wt.% for F357 (Al-7Si-0.65 Mg) casting alloy. Kim et al. [22] also found that 0.8 wt.% Sc addition was optimal content for Al-8.5Si alloy. In general, the optimal addition of Sc required for different Al-Si-Mg casting alloys varies significantly with different Si and Mg contents. However, the determination of the optimal Sc addition for different Al-Si-Mg casting alloys by the experimental trial-and-error method is not only time/labor-consuming but also imprecise. What’s more, it is almost impossible to accurately explore the optimal contents of Si, Mg, and Sc in hypoeutectic Al-*x*Si-*y*Mg-*z*Sc casting alloys over high-dimensional composition space by the trial-and-error experiments. Therefore, there is an urgent need to remedy this situation.

With the development of computer technology, various computational assisted alloy design methods, like first-principles (FP) calculations [33], molecular dynamics (MD) simulations [34], computational thermodynamics (CT) [11,15], computational kinetics [35,36], phase-field (PF) simulations [37], and machine learning (ML) approach [38,39], have been widely used to accelerate the development of high-performance alloys. The CT method, which can construct the quantitative relationship between the composition and microstructure of alloys, has been recently used to efficiently design the optimal Sc contents in i.e. A356 (0.54 wt.% Sc), A357 (0.50 wt.% Sc), A360 (0.76 wt.% Sc), and A380 (0.75 wt.% Sc) alloys, and also experimentally validated [11,15]. Furthermore, the combination of CT and ML techniques, which can establish the quantitative relation ‘composition-process-microstructure-properties’ of target alloys and thus accelerate the design of alloy composition, has been employed to efficiently design the optimal addition of Sr in A356 alloy [40]. Thus, such a combination of CT and ML techniques may stimulate the efficient composition design in multicomponent alloys [41,42].

However, there are still big challenges when one directly applies such a combination of CT and ML techniques to optimize the contents of Si, Mg, and Sc in the present Al-*x*Si-*y*Mg-*z*Sc alloys. The first big challenge lies in that a large number of datasets are needed to establish the quantitative relation ‘composition-process-microstructure-properties’ of Al-*x*Si-*y*Mg-*z*Sc alloys over high-dimensional composition space. For the casting Al-Si-Mg alloys, it is well known [41,43] that one may obtain a vast amount of data for reliable relation ‘composition-process-microstructure’ by combining the high-throughput CALculation of PHAse Diagrams (CALPHAD) simulations with key experiments. However, there are too limited experimental data in the literature to develop the quantitative relation 'microstructure-properties'. That is because most of the current data devote to the Sc-modified A356 alloys, while for other commercial alloys, i.e. A357 and A360, the experimental data are very scarce, not to mention the alloy compositions that do not belong to the commercial alloys. The second big challenge is how to achieve the multi-objective optimization, including ultimate tensile strength (UTS), yield strength (YS), and elongation (EL), of the Al-Si-Mg-Sc casting alloys.

For the first challenge, based on the ocean of CALPHAD data on ‘composition-process-microstructure’ and limited experimental data on mechanical properties, the active learning technique can be applied and may result in designing the next experimental points by the CALPHAD and Bayesian optimization samplings, experimental validation, and data feedback iterations, which can reduce the uncertainty of models and improve the predicted accuracy with the least number of iterations. Such a strategy should be beneficial to efficiently construct the required dataset with a minimum size. As for the second challenge, the multi-objective optimization strategies, like the sequential filter strategy [44], the transformation of multi-objective into single-objective optimization methods [45], Pareto front optimization method [46], can be utilized.

Consequently, in this paper, a novel alloy design strategy integrating activating learning with high-throughput CALPHAD calculations and key experiments is proposed to accurately explore the optimal contents of Si, Mg, and Sc in hypoeutectic Al-*x*Si-*y*Mg-*z*Sc casting alloys over high-dimensional composition space with the multi-objective optimization. The quantitative relation ‘composition-process-microstructure’ of hypoeutectic Al-Si-Mg-Sc casting alloys is first established by the high-throughput CALPHAD solidification simulations. Then, the reliable relation ‘microstructure-properties’ of hypoeutectic Al-Si-Mg-Sc casting alloys is acquired using the active learning technique supported by key experiments designed by CALPHAD and Bayesian optimization samplings. After that A356-*x*Sc alloy system is chosen as the benchmark, and the present strategy is utilized to design the high-performance hypoeutectic Al-*x*Si-*y*Mg alloys with optimal Sc additions and then validated by experiments. Finally, the present strategy is extended to screen the optimal contents of Si, Mg, and Sc over high-dimensional hypoeutectic Al-*x*Si-*y*Mg-*z*Sc composition space.

## 2. Methods

### 2.1. Design strategy

**Figure 1.**
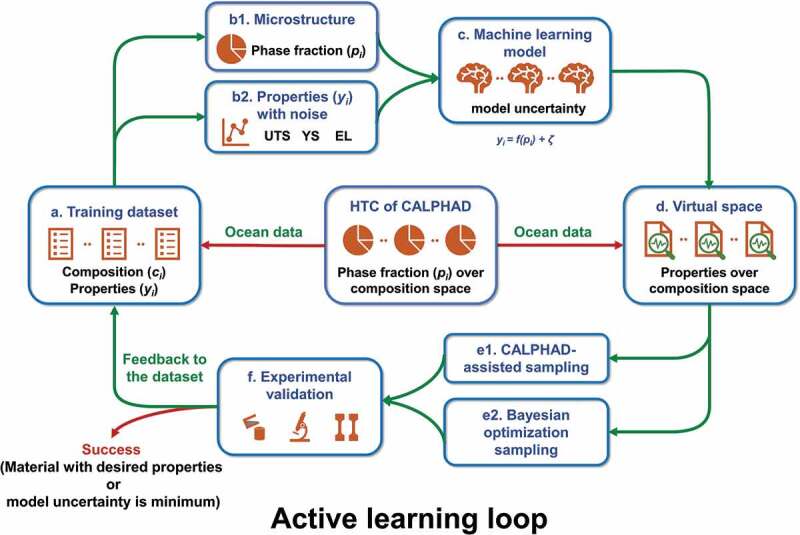
Schematic diagram for the presently proposed alloy design approach for accelerating the discovery of multi-component alloy by integrating the active learning with HTC of CALPHAD and key experiments. High-throughput CALPHAD simulations are used to construct the relation of ‘composition/process-microstructure’ for multi-component casting alloy and provide an ocean of data for the predicted dataset of machine learning model. Machine learning model is used to establish the relation of ‘microstructure-properties (UTS, YS, EL)’ for multi-component alloy. Active learning strategy is used to accelerate the discovery of high-performance multi-component alloy systems over high-dimensional composition space.

The schematic diagram for our multi-component alloys discovery strategy is shown in Figure 1. Firstly, the literature data were collected to construct the training dataset, which includes the composition and mechanical properties. The corresponding solidified microstructures (type and fraction of solidified structures) were calculated by high-throughput Scheil-Gulliver solidification simulations based on the reliable thermodynamic database of Al-Si-Mg-Sc system [11], from which the relation of ‘composition-process-microstructure’ was established, and used as the input features. Then, the features were sorted and analyzed to determine the relationship between the input features and the impact on output features (properties), i.e. UTS, YS, and EL. The artificial neural network (ANN) was used to construct the relation of ‘microstructure-properties’ from low- to high-dimensional composition space. The uncertainties from the data noise, data source, and trained state of the model were considered to evaluate the predicted results. Moreover, the input microstructural features space over a wide composition range of hypoeutectic Al-Si-Mg-Sc casting alloys was constructed by the high-throughput CALPHAD solidification simulations, and used to predict the properties over the wide composition space. The initial experimental dataset was constructed by CALPHAD-assisted sampling. After considering the uncertainties of machine learning prediction, Bayesian optimization was employed to perform the globally efficient design of the next experimental points with the best properties over the searching space. After that, the designed Sc-modified Al-Si-Mg casting alloys were prepared and the mechanical properties were measured to validate the design results, and feedback to the training dataset for the next iteration. Besides, some stopping criteria were applied to avoid pointless over-exploration. For instance, the iteration should stop when the result meets the expectations or when the improvement of uncertainty is not noticeable [47]. Finally, the mechanical properties of hypoeutectic Al-Si-Mg-Sc casting alloys over the wide composition space were obtained with an efficient strategy, and the high-performance hypoeutectic Al-Si-Mg-Sc casting alloys were recommended.

### 2.2. High-throughput CALPHAD calculations

Up to now, the CALPHAD approach has been widely used in the design and development of different high-performance materials, but the only CALPHAD computational tool is generally of low efficiency in exploring the entire composition and temperature space of a multi-component system [48]. In order to meet the increasing demand for massive calculation in the field of high-performance aluminum alloy, a machine learning accelerated distributed task management system (Malac-Distmas) has been developed in our research group to realize high-throughput calculations (HTCs) and storage of various data [43].

With the platform Malac-Distmas coupling Thermo-Calc software [49], we can perform HTC of Scheil-Gulliver simulation for Sc-additional Al-Si-Mg casting alloy over the commercial hypoeutectic composition space, to obtain their solidified microstructure and construct the solidification diagram. During the calculations, the content range of Si was set between 4.5 and 13 wt.% with ∆*w*(Si) = 0.5 wt.%, that of Mg was between 0 and 0.7 wt.% with ∆*w*(Mg) = 0.05 wt.%, while that of Sc was between 0 and 1 wt.% (when *w*(Si)≤10 wt.%) or between 0 and 1.3 wt.% (when *w*(Si)>10 wt.%) with ∆*w*(Sc) = 0.01 wt.%. The calculation range of Sc content mainly depends on the Si content, while the optimal Sc content designed from the solidification diagram increases with the increase of Si content. It means that 29,519 composition points of Scheil-Gulliver simulations need to be calculated, and it is almost impossible to perform so many simulations only by manually submitting the simulation tasks. With a client program of Malac-Distmas, 101 tasks of the Scheil-Gulliver simulations can be completed within 20 min using Thermo-Calc software. It costs 4 days with Malac-Distmas and two Thermo-Calc clients to finish the present simulations. The simulation results were extracted and saved in an SQL-format database with a total size of   20 MB.

### 2.3. Machine learning technique

Machine learning modeling aims to establish a function between input and output and makes it as close to the real function relationship as possible by optimizing the model parameters [50,51]. Due to its low computational cost and short development cycle, machine learning is coupled with powerful data processing and high prediction performance and is being widely used in material science, including the establishment of phase diagrams [52], properties prediction [53], the discovery and design of high-performance materials [38,54], and the exploration of strengthening and toughening mechanism [38,40]. The widely used machine learning algorithms include linear algorithms, decision tree-based (DT) algorithms, artificial neural network (ANN), support vector machines (SVM), random forest (RF), and some Bayesian-based algorithms [55]. ANN is the most common approach in machine learning and will be used in this work. The open-source platforms for machine learning, Scikit-learn [56] and Pytorch [57], were used in this work.

### 2.4. Active learning

When the sample data are scarce due to the experimental challenges or high costs, it is difficult to establish a machine learning model with high prediction accuracy and great generalization ability using an existing small dataset. Hence, the active learning method that uses the designed experimental iterative feedback optimization method to improve the machine learning model predictions and reduce the number of required experiments has attracted attention [50,58]. Results of Lookman et al. indicate that active learning is forgiving of poor model quality [47]. The active learning method includes the following steps: data collection, feature engineering, machine learning model construction, next experimental points design, experimental test, and data feedback. The key point of active learning is to design the next experimental points according to the predicted value and prediction uncertainty of the machine learning model, and then iteratively optimize the next modeling by the feedback of experimental results until the model prediction meets the requirements [59].

In materials design, the common sampling strategies include manual empirical sampling, CALPHAD-assisted sampling, and Bayesian optimization sampling. Bayesian optimization (BO), which is an efficient global optimization method based on adaptive sampling, is one of the most common experimental points design methods in material exploration [47,60,61]. Bayesian optimization method evaluates the utility or acquisition function based on the model’s predicted mean value and prediction uncertainty to design the next experimental points. The utility function allows a balance between exploitation (sampling where the objective mean is high) and exploration (sampling where the uncertainty is high). Among the available utility functions (i.e. Probability of Improvement (PI), Expected Improvement (EI), and Expected Upper Confidence Bounds (UCB)), the EI, which considers not only the probability of improvement but also the expected magnitude of improvement, is the widely used as utility function [62]. The EI [63] is defined as

(1) $$EI(x)=\left\{\begin{array}{c} \left(\mu (x)-f(x^{+})-\xi \right)\Phi \left(\frac{\mu (x)-f(x^{+})-\xi }{\sigma (x)}\right)\\ \;\;\;\;\;\;\;\;\;+\sigma (x)\phi \left(\frac{\mu (x)-f(x^{+})-\xi }{\sigma (x)}\right)\;\;\;\;\;\;\;if\;\sigma (x)>0\\ 0\;\;\;\;\;\;\;\;\;\;\;\;\;\;\;\;\;\;\;\;\;\;\;\;\;\;\;\;\;\;\;\;\;\;\;\;\;\;\;\;\;\;\;\;if\;\sigma (x)=0 \end{array}\right.$$

where *μ* and *σ* are the mean and the standard deviations of objective function *f*(*x*), respectively. *f*(*x*^+^) is the value of the best sampling so far and *x*^+^ is the location of that sample. Φ(•) and *ϕ*(•) are the cumulative distribution function (CDF) and the probability distribution function (PDF) of the standard normal distribution. Parameter ***ξ*** can be used to further control the trade-off between global search and local optimization, and determines the amount of exploration during optimization and higher ***ξ*** values lead to more exploration. A recommended default value for ***ξ*** is 0.01 [63]. Thus, a new sample point, x*, is chosen amongst other data points based on the largest EI, i.e. x* = *argmax*_*x*_EI(*x*).

For quantifying uncertainties, three types of uncertainties should be considered: *i*) the experimental uncertainty, which corresponds to the noise observations of the same one batch experiments; *ii*) the data uncertainty or data noise, which corresponds to the sparse data and data from various sources, especially for the experimental data, and *iii*) the model uncertainty, which includes the model selection and random state of one model [64]. A confidence interval in the model prediction can be drawn by identifying and quantifying the sources of the uncertainty. This not only allows users to understand the prediction reliability but also facilitates the implementation of active learning [65,66].

Therefore, the experimental noise (error) of Sc-modified Al-Si-Mg casting alloys was considered with the Gaussian distribution function in this work. Moreover, the mean and standard deviation of the tensile mechanical properties of Sc-modified Al-Si-Mg casting alloys with the same composition from different sources were re-evaluated with different weights that depend on the ratio between the respective number of experiments and the total number, as shown in Table 1. The ANN model including one hidden layer with 15 neurons was used in this work and all hyperparameters were fixed except the initial training state to avoid the effect of other hyperparameters on uncertainties. For each feature group, the ANN model was repeated 1000 times with the initial training state from 0 to 999 with and without the experimental data noise (error), resulting in 1000 ANN models for evaluating the uncertainty. The ‘leave-one-out’ method was used to validate the models. An ensemble of these 1000 models then gave predictions with means *μ* and standard deviation *σ*. Materials with the highest EI were selected for preparation.

**Table 1.** List of normal composition and tensile properties of Sc-modified Al-Si-Mg casting alloys.

Iteration group	Alloy	Si	Mg	Sc	*W*_i_^**^	UTS (MPa)	YS (MPa)	EL (%)	Ref
1^st^	A356	6.9	0.44	0	1	201 ± 4	106 ± 2	4.6 ± 0.2	[11]
	A356–0.21Sc	6.9	0.44	0.21	1	213 ± 3	128 ± 7	4.6 ± 0.1	
	A356–0.41Sc	6.9	0.44	0.41	1	235 ± 5	148 ± 4	5.4 ± 0.2	
	A356–0.65Sc	6.9	0.44	0.65	1	239 ± 3	149 ± 6	3.9 ± 0.3	
	A356–0.75Sc	6.9	0.44	0.75	1	238 ± 5	148 ± 4	3.6 ± 0.2	
	A356–0.54Sc*	6.9	0.44	0.54	6/8	239 ± 3	153 ± 2	5.3 ± 0.1	
	A356–0.54Sc*	6.9	0.44	0.54	1/8	232 ± 3	136 ± 3	8.0 ± 1.0	[16]
	A356–0.54Sc*	6.9	0.44	0.54	1/8	231 ± 3	135 ± 4	6.9 ± 0.4	This work
2^nd^	A357	7	0.6	0	1	213 ± 4	122 ± 2	5.1 ± 0.3	[15]
	A357–0.53Sc*	7	0.6	0.53	2/3	238 ± 5	158 ± 4	5.4 ± 0.2	
	A357–0.53Sc*	7	0.6	0.53	1/3	236 ± 3	142 ± 3	5.9 ± 0.3	This work
	A360	9.5	0.5	0	1	226 ± 6	127 ± 4	4.8 ± 0.3	[15]
	A360–0.79Sc*	9.5	0.5	0.79	2/3	273 ± 4	158 ± 5	6.4 ± 0.1	
	A360–0.79Sc*	9.5	0.5	0.79	1/3	243 ± 2	138 ± 3	5.0 ± 0.2	This work
	A380	8.5	0	0	1	161 ± 2	63 ± 4	10.2 ± 0.3	[15]
	A380–0.77Sc*	8.5	0	0.77	2/3	212 ± 4	114 ± 3	10.0 ± 0.1	
	A380–0.77Sc*	8.5	0	0.77	1/3	202 ± 4	100 ± 3	7.3 ± 0.2	This work
3^rd^	355–0.35Sc*	5	0.5	0.35	1	215 ± 4	126 ± 4	8.7 ± 0.2	This work
	A413–1.14Sc*	12	0	1.14	1	214 ± 3	99 ± 5	7.8 ± 0.1	
	359–0.73Sc*	9	0.5	0.73	1	245 ± 4	140 ± 3	5.8 ± 0.3	
4^th^	A1*	13	0.7	1.11	1	253 ± 4	152 ± 3	2.3 ± 0.2	This work
5^th^	A2*	9.5	0.7	0.75	1	241 ± 4	142 ± 3	5.0 ± 0.1	This work

*The Sc content is optimal for the hypoeutectic Al-*x*Si-*y*Mg casting alloy based on the results of high-throughput CALPHAD solidification simulations.

^**^The weight *Wi* is used to re-calculate the mean and standard deviations of mechanical properties for the alloys with the same composition. Moreover, the weights depend on the ratio between the respective number of experiments and the total number. For example, for A356–0.54Sc alloy, the weight of data from Ref. [11] is 6/8 (0.75), while is 1/8 (0.125) for the data from Ref. [16] and this work.

### 2.5. Experimental procedure

To avoid the effect of minor element contamination, the Al-Si-Mg master alloys were prepared using the high-purity elements with purity up to 99.99 wt.% purchased from Alfa Aesar (China) Chemicals Co., Ltd. Since magnesium evaporates easily during melting, an amount of extra 5 wt.% magnesium was added in each sample. Each alloy sample was melted in a graphite crucible using an inductive furnace under an argon gas atmosphere. After homogenizing for 5 min at the temperature of 720°C, the Al-2Sc was added at 750°C to obtain the required chemical composition, as listed in Table 1. Each sample was then cast into a cylindrical graphite mold with a diameter of 20 mm and a height of 150 mm and preheated to 100°C. A universal testing machine (Instron 3369, USA) with a loading speed of 1 mm/min was used to test the room-temperature tensile of the alloys. For each alloy, four specimens were tested, and the mean values were accepted.

## 3. Results and discussion

### 3.1. High-throughput Scheil solidification simulations

Based on the solidification diagram constructed with a huge amount of Scheil solidification simulations, Lu et al. [11] from our research group designed the optimal Sc content (i.e. 0.54 wt.%) in A356 with the criterion that the binary eutectic structure was completely replaced by the ternary eutectic structure, and their experimental result also showed that A356–0.54Sc alloy owned the best mechanical properties over the composition range of A356-*x*Sc. In addition, Lu et al. [15] designed the optimal Sc contents in A357 (0.50 wt.% Sc), A360 (0.76 wt.% Sc), and A380 (0.75 wt.% Sc) alloys based on the solidification diagram, and their experiments nicely validated their predictions. Therefore, the CALPHAD-assisted alloy design method can be used to establish the relation of ‘composition-process-microstructure’ for efficiently exploring the high-performance Sc-modified hypoeutectic Al-Si-Mg casting alloys.

**Figure 2.**
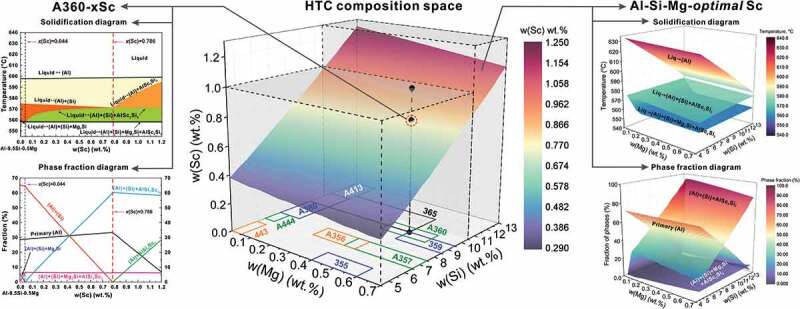
Results of high-throughput CALPHAD solidification simulations. Composition space forces on the commercial hypoeutectic casting Al-Si-Mg-Sc alloys: *w*(si)∈[4.5,13] wt.%, *w*(mg)∈[0, 0.70] wt.%, and *w*(sc)∈[0,1] wt.% when w(si) ≤ 10 wt.%, and *w*(sc)∈[0,1.3] wt.% when w(si) > 10 wt.%. High-throughput Scheil-Gulliver solidifications were performed. Each vertical line represents a Sc-modified Al-Si-Mg alloys series, i.e. A360-*x*Sc alloys, and the corresponding solidification diagram and phase fraction diagram can be constructed to design the optimal Sc content (i.e. 0.79 wt.% for A360 alloy) based on the criterion that binary eutectic structure was completely replaced by the ternary eutectic structure. Moreover, the optimal Sc content for each Al-Si-Mg hypoeutectic casting alloy can be designed with a similar criterion and plotted as a plane. The corresponding solidification diagram and phase fraction diagram of this plane were shown on the right side.

Figure 2 displays the partial results for high-throughput Scheil-Gulliver solidification simulations of Sc-modified commercial hypoeutectic casting Al-Si-Mg alloys, of which the concentration ranges of Si and Mg are in [4.5, 13] wt.% and [0, 0.7] wt.%, respectively. The composition ranges of commercial hypoeutectic casting Al-Si-Mg alloys [67], i.e. A356, A357, 359, A360, 365, A380, A413, 443, A444, and so on, were plotted on the horizontal projection plane. Each vertical line represents a Sc-modified Al-Si-Mg alloy series, such as A360-*x*Sc alloys shown on the left side of Figure 2, and the corresponding solidification diagram and phase fraction diagram can be constructed to design the optimal Sc content (0.79 wt.%) in A360 alloy with the criterion that the binary eutectic structure was completely replaced by the ternary eutectic structure.

Besides the optimal Sc content plane of the commercial hypoeutectic Al-Si-Mg casting alloys, the corresponding solidification diagram and phase fraction diagram of alloys in this plane were shown on the right side of Figure 2. As can be seen from the solidification diagram of Al-*x*Si-*y*Mg-*optimal* Sc alloys, when Mg content is above 0 wt.%, the alloys with optimal Sc contents have the same solidified sequence with the pre-set criteria, i.e. that liquid→(Al), liquid→(Al)+(Si)+AlSc_2_Si_2_, and liquid→(Al)+(Si)+AlSc_2_Si_2_+Mg_2_Si. When Mg content equals 0 wt.%, the alloys with optimal Sc content have the solidified sequences as liquid→(Al), liquid→(Al)+(Si)+AlSc_2_Si_2_. With the increase of Si content, the melting point of alloys decreases, as shown in the solidification diagram of Al-Si-Mg-*optimal* Sc alloys. The temperature of quaternary eutectic reaction (Liquid→(Al)+(Si)+AlSc_2_Si_2_+Mg_2_Si) remains constant (558.22°C) in the Sc-additional Al-Si-Mg casting alloys, and the temperature surface is thus a plane. Moreover, with the increase of Si content, the fraction of primary (Al) decreases, and the fraction of ternary eutectic structure increases.

### 3.2. Benchmark in Sc-modified A356 alloys

The relationship between the alloy features and experimental properties can be constructed by machine learning and can be used to design alloys with better properties. Moreover, the input determines the output for machine learning. Good features might be more important than the optimization of model hyper-parameters [68–70]. Selecting appropriate features can not only reduce prediction overfitting, improve the signal-to-noise ratio, prevent dimensional disaster, and improve prediction generalization ability, but also make the model better interpretability.

In most research about machine learning-assisted alloy design, the common input features are compositions of different components [53,71]. However, with the high-throughput CALPHAD solidification simulations, the solidified microstructure of Sc-modified commercial hypoeutectic Al-Si-Mg casting alloys can be obtained and used as features. Accordingly, the effect of the different features on machine learning results in the Sc-modified A356 casting alloys will be investigated. Furthermore, the uncertainties from the data noise and trained state of the machine learning model are quantified.

**Figure 3.**
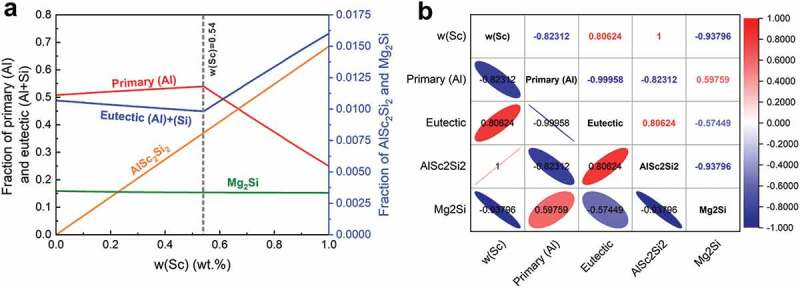
Features analysis of A356-*x*Sc alloys. (a) Solidified microstructure features of A356-*x*Sc alloys, (b) Pearson correlation coefficient of the solidified microstructure features of A356-*x*Sc alloys.

Based on the simulated microstructure (as shown in Figure 3(a)) and the experimental results of A356-*x*Sc casting alloys except for the composition point (0.54 wt.% Sc) with best properties [11] listed in Table 1, two feature groups of A356-*x*Sc alloys were set: *i*) the traditional feature group A, in which the input feature is the Sc content while the output features are the properties (UTS, YS, EL), *ii*) the microstructural feature group B, in which the input features are the calculated fractions of primary (Al) phase, total eutectic (Al)+(Si), AlSc_2_Si_2_ phase, and Mg_2_Si phase, as validated by the experiments [11], while the output features are the same with feature group A. For microstructural features (as shown in Figure 3(a)), the fraction of primary (Al) phase firstly increases with the increase of Sc content and decreases when *w*(Sc) is over 0.54 wt.%. The change of eutectic (Al)+(Si) is the opposite of that of the primary (Al). The fraction of AlSc_2_Si_2_ phase increases as the Sc content increases, but the fraction of Mg_2_Si remains the same. Besides, the Pearson correlation coefficient was used to analyze the linear relation among these features, as shown in Figure 3(b). The results also show that the fraction of eutectic (Al)+(Si) structure is a highly negative linear relationship with the fraction of primary (Al) in the microstructural feature group. In principle, one microstructure feature can be removed in such two strongly linearly correlated features to achieve the feature dimension reduction for better model performance. However, we retain all the microstructural features, and there are two reasons: *i*) that the comparison between the simulated phase fractions and the experimental data is facilitated; and *ii*) that the effects of each feature on the mechanical properties of alloys can be analyzed, and the strengthening/toughening mechanisms can be then discussed.

**Figure 4.**
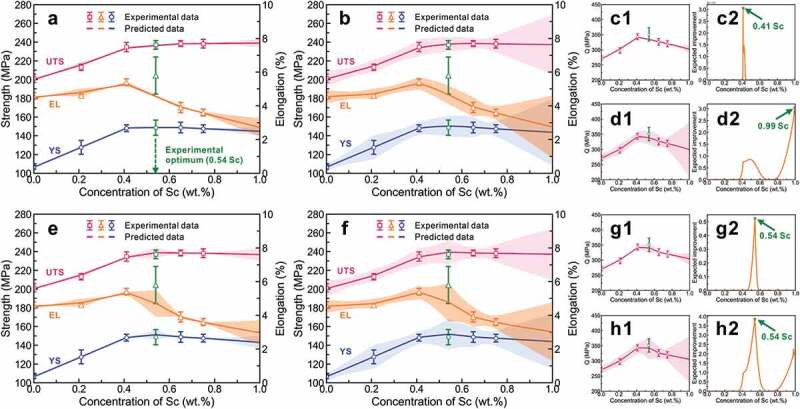
Machine learning-assisted design for A356-*x*Sc alloys. 1000 ANN models results with two feature groups for experimental properties of A356-*x*Sc [11] excluding the best points: (a–d) Features group A: *w*(sc), (e–h) Features group B: *f*(fcc), *f*(eutectic (Al)+si), *f*(AlSc_2_Si_2_), *f*(Mg_2_Si). (a,c,e,g) for data without noise, (b,d,f,h) for data considering noise. The comprehensive mechanical property was calculated using the quality index *Q*=*UTS*+*YS*•log_10_(*EL*) in (c1,d1,g1,h1), and the EI value was evaluated to design the next point in (c2,d2,g2,h2). The error ranges of predicted results are plotted with 2 standard deviations of the mean value of 1000 ANN model results, corresponding to the confidence interval of 95%.

Figure 4 shows that the plot with the green color of the 1000 ANN model results in two feature groups for A356-*x*Sc excluding the best point (0.54 wt.% Sc). The error ranges of predicted results are plotted with two standard deviations of the mean value of 1000 ANN model results, corresponding to the confidence interval of 95%. When the experimental mechanical properties noises were not considered, the uncertainty of results was only contributed from the initial training state of the machine learning model, as shown in Figure 4(a,e). The results show that the training results describe well the existing training data, and the uncertainty from the initial training state of the machine learning model is small. However, the results from feature group A suggest that it is impossible to predict the optimal experimental elongation, while the results from feature group B show that there is a great probability to obtain the optimum properties at 0.54 wt.% Sc. Moreover, in order to perform the efficient global design of the next experimental points based on the trained results, the multi-objective (UTS, YS, EL) is transformed into a single-objective to represent the comprehensive mechanical property by using the quality index *Q*=*UTS*+*YS*•log_10_(*EL*), which was slightly modified based on *Q*_*DJR*_ = *UTS* + c•log_10_(*EL*) [72] by replacing the constant value c (i.e. ca. YS value of A356 alloy) by the composition-dependent YS values over the wide composition range in this work. Then, the EI can be then calculated using Equation (1) with the mean and standard deviation of *Q* and used to determine the next point with maximum EI. The results of the comprehensive mechanical property of the two feature groups without data noise are presented in Figure 4(c1,g1). As indicated in the figures, there is a great probability to obtain the optimal experimental comprehensive mechanical property when the feature group B is used. The corresponding EI values are shown in Figure 4(c2,g2), and the designed next points with maximum EI values from the models with feature group A and feature group B are 0.41 and 0.54 wt.% Sc, respectively. The results show that models with feature group B (the microstructural features) are more efficient and accurate to design the optimal experimental point than those with feature group A (the composition features).

Furthermore, the data noises with Gaussian distribution function for experimental mechanical properties were considered in 1000 ANN models with different initial training states. The training results of two feature groups with data noise are presented in Figure 4(b,f). It can be seen that the errors of the experimental data are within the confidence interval of prediction. For one feature group, the results without and with data noise suggest that the uncertainty from data noise is larger than that from the initial training state models. However, in terms of predicting the optimal experimental points, the results are the same as those without considering data noise. The corresponding quality index *Q* and EI were calculated based on the predicted results, as shown in Figure 4(d1-d2,h1-h2). As can be seen from Figure 4(d1,d2), although the *Q* results of the models with feature group A and considering data noise can cover a part of experimental optimal *Q*, the designed next point with maximum EI is 0.99 wt.% Sc, which is already far from the experimentally optimal point. The models with feature group B and considering data noise not only describe the *Q* results of training experimental data well but also predict and design the experimental optimal point, which can be validated by the results of EI, as shown in Figure 4(h1,h2). Compared with feature group A, feature group B is more suitable in ANN models for describing the experimental data, and the corresponding training results can accurately design the next experimental point, especially when the experimental error is taken into account. The main reason for the difference might be that there is only one monotonic variable (Sc content) as an input feature in feature group A, while the properties of the material are closely related to the microstructure used in feature group B. Therefore, feature group B (the microstructural features) will be selected in this work and the uncertainties are quantified from models and data noise.

### 3.3. Discovery of hypoeutectic Al-xSi-yMg-optimal Sc alloys

Combining CT and ML techniques, the quantitative relationship of ‘composition-process-microstructure-property’ of Sc-modified hypoeutectic Al-Si-Mg casting alloys can be established to efficiently design the optimal Sc content, such as A356-*x*Sc alloys. However, it is foreseeable that there is considerable uncertainty in predicting the mechanical properties of hypoeutectic Sc-modified Al-Si-Mg casting alloy using the models only based A356-*x*Sc data. Figure 6(a) shows the trained result of the comprehensive mechanical property index *Q* with the experimental data of A356-*x*Sc. The result of 1^st^ iteration shows that there is a large uncertainty when predicting the properties of Al-*x*Si-*y*Mg-optimal Sc. In order to accurately explore the optimal mechanical properties of commercial hypoeutectic Al-Si-Mg-Sc casting alloys, more data are needed to improve the ML models. The active learning method, which uses the designed experimental iterative feedback optimization method to improve the machine learning model predictions and reduce the number of required experiments, was utilized in this work.

**Figure 5.**
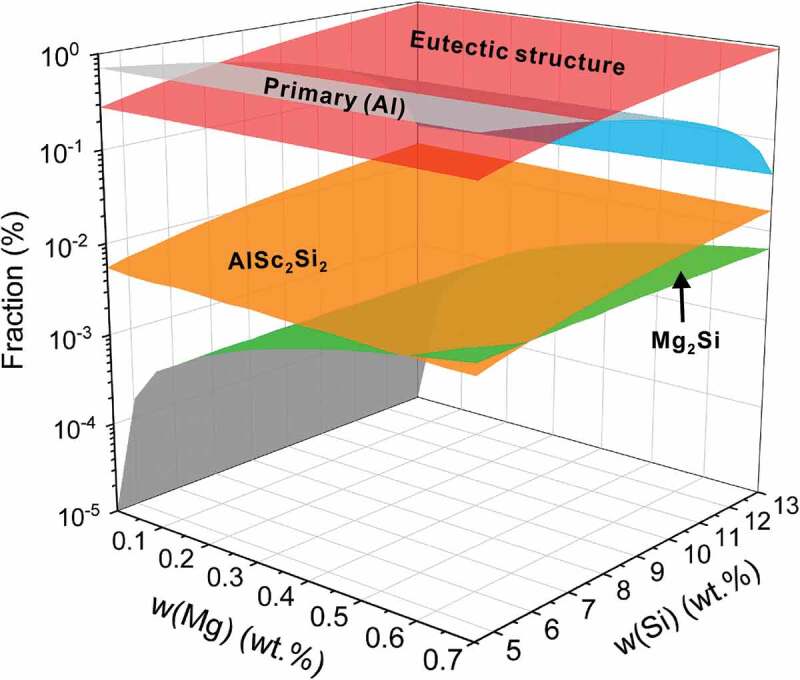
Microstructural features of the hypoeutectic Al-*x*Si-*y*Mg-optimal Sc casting alloys. Microstructural features, which are selected based on the benchmark results in Sc-modified A356 alloys, are extracted from the high-throughput solidification simulations of the hypoeutectic Al-Si-Mg-Sc casting alloys.

**Figure 6.**
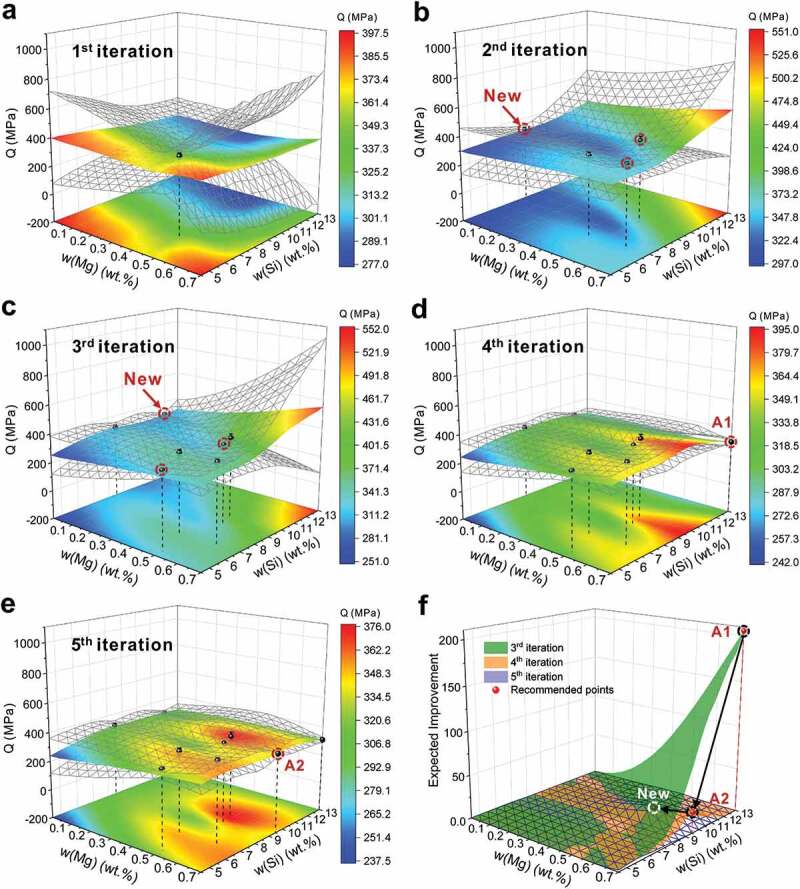
Active learning results of hypoeutectic Al-*x*Si-*y*Mg-optimal Sc casting alloys. Predicted results of ANN models and experimental comprehensive mechanical property index *Q* for the hypoeutectic Al-Si-Mg alloys with optimal Sc content during active learning processes. (a–c) initial dataset was constructed by CALPHAD-assisted sampling. (b–e) Bayesian optimization sampling based on the EI in (f). The gray surfaces represent the 95% confidence interval of the predictions.

The key to the improvement of ML models is the sampling strategy. The common sampling strategies include manual empirical sampling, CALPHAD-assisted sampling, and Bayesian optimization sampling. For the construction of the initial training dataset, manual empirical sampling is specially considered. Fortunately, CALPHAD-assisted sampling (CT method) is an efficient method to accurately design alloy. Based on the alloy design results of Sc-modified A356 casting alloys, the CT method can be used to efficiently design the optimal Sc addition for hypoeutectic Al-Si-Mg casting alloys with the optimal mechanical properties and the optimal Sc addition over the composition space of hypoeutectic Al-Si-Mg casting alloys has been performed based on the high-throughput Scheil solidifications (as shown in Figure 2) and can be described by Eq. (S1) in supplementary materials. Hence, the high-performance composition searching space for hypoeutectic Al-Si-Mg-Sc casting alloys was forced on the optimal Sc content surface, and the corresponding microstructural features used in the ANN models are shown in Figure 5. As can be seen from Figure 5, the fraction of the total eutectic (Al)+(Si) structure and AlSc_2_Si_2_ phase increase obviously with the increasing Si content, while the change of primary (Al) phase is opposite to the change of the total eutectic (Al)+(Si) structure. The amount of Mg_2_Si is relatively small and its variation is mainly related to the Mg content. The previous 1000 ANN models considering the data noise were iteratively optimized with the feedback of new experimental points. The sampling process was conducted in the following two steps:

**Step 1: CALPHAD-assisted sampling**. In order to accurately explore the optimal mechanical properties of commercial hypoeutectic Al-Si-Mg-Sc casting alloy, more data were chosen for the commercial hypoeutectic Al-Si-Mg alloys with the optimal Sc content designed based on the CT method. As shown in Table 1, the new experimental data during the 2^nd^ iteration were from Lu’s work in 2019 years (A357 and A357–0.53Sc, A360 and A360–0.79Sc, A380 and A380–0.77Sc), which are on the Al-*x*Si-*y*Mg-optimal Sc surface. The weights based on the proportion of the number of sampling in this system were used to calculate the mean and standard deviation of mechanical properties for the alloys with the same composition. What’s more, the new experimental data were added into the 3^rd^ iteration, including the 355–0.35Sc, A413–1.14Sc, and 359–0.73Sc from this work. Figure 6(a–c) present the quality index *Q* for experimental comprehensive mechanical property and the predicted results after 3 iterations for the optimal Sc-modified hypoeutectic Al-Si-Mg alloys (the solidified features and the training results of A357-*x*Sc, A360-*x*Sc, and A380-*x*Sc can be seen in Supplementary Figures S2 and S3). It should be noted that the gray surfaces represent the 95% confidence interval of the predictions. As shown in Figure 6(a–c), the predictions of ANN models describe the experimental results well. As new experimental data were fed back into the models for iterative optimization, the prediction of ANN models was improved and the uncertainty around the experimental data was reduced. The ML models describing the relationship between the microstructure and properties for the hypoeutectic Al-Si-Mg casting alloys with optimal Sc were preliminarily established. Furthermore, in this composition space of hypoeutectic Al-Si-Mg casting alloys with optimal Sc content, the predicted results show that the composition with the best comprehensive mechanical property index *Q* is located in the corner of the space with maximum Si and Mg content, accompanied by a large uncertainty.

However, there is no commercial hypoeutectic Al-Si-Mg series corresponding to the composition with maximum *Q*. It is difficult to further design the next points only based on the CALPHAD-assisted sampling strategy. Bayesian optimization sampling, which is an efficient global optimization method based on adaptive sampling, was then applied to design the new experimental points and further reduce the uncertainty of the models.

**Step 2: Bayesian optimization sampling**. Based on the iterative results of the previous step, the EI over the composition space of optimal Sc-modified hypoeutectic Al-Si-Mg alloys, allowing a balance between exploitation (sampling where the objective mean is high) and exploration (sampling where the uncertainty is high), was calculated using Equation (1) and shown in Figure 6(f). As can be seen from Figure 6(f), after the 3^rd^ iteration, the maximum EI is located in the corner of the space with maximum Si and Mg content (13 wt.% Si and 0.7 wt.% Mg with 1.11 wt% optimal Sc), and value of EI is 206. Thus, considering that the next point is designed based on *max*(EI), the corner point (Al-13Si-0.7 Mg-1.11Sc) with predicted *Q* = 552 ± 240 MPa is chosen as the next point, marked as A1. Then, the A1 alloy was prepared and the mechanical properties were measured, as listed in Table 1. The experimental results show that UTS, YS, and EL of A1 alloy were 253 ± 4 MPa, 152 ± 3 MPa, and 2.3 ± 0.2%, respectively, and the *Q* is 308 ± 10 MPa and at the low boundary of the predicted range from the 3^rd^ iteration. Afterward, the experimental properties of Al alloy were fed back to the dataset as the 4^th^ iteration for optimizing the models, and the iterative results are shown in Figure 6(d). Compared with the results of the 3^rd^ iteration in Figure 6(c), the prediction accuracy and uncertainty of models after the 4^th^ iteration were significantly improved over the entire concerned composition space. The EI after the 4^th^ iteration was calculated and shown in Figure 6(f). The results show that the EI of models after the 4^th^ iteration reduced significantly and the maximum EI was 24.25 at the Al-9.5Si-0.7 Mg (optimal Sc 0.75 wt.%), which is located in the maximum Mg boundary and the boundary of composition windows of commercial 359 alloys (Si:8.5–9.5 wt.%, Mg: 0.5–0.7 wt.%). Therefore, Al-9.5Si-0.7 Mg-0.75Sc alloy marked as A2 with predicted *Q* = 395 ± 40 MPa, as the designed alloy of the 4^th^ iteration, was also prepared and compared with the 359–0.73Sc alloy in the 3^rd^ iteration to validate and feedback optimize the models.

The mechanical properties of A2 alloy were measured as listed in Table 1. The experimental results showed that UTS, YS, and EL of A2 alloy were 241 MPa, 142 MPa, and 5.0%, respectively, and the *Q* was 340 ± 7 MPa. The experimental *Q* of A2 alloy falls out of the low boundary of predicted *Q* from the 4^th^ iteration. However, the measured mechanical properties of A2 are quite similar to those of existing 359–0.73Sc alloy (UTS: 245 ± 4 MPa, YS: 140 ± 3 MPa, EL: 5.8 ± 0.3%, *Q*: 352 ± 9 MPa).

The experimental properties of A2 were fed back to the dataset as iteration 5 for optimizing the models, and the iterative results were updated and given in Figure 6(d). After the 5^th^ iteration, the predicted accuracy of models was improved slightly, and the alloy composition space with the highest *Q* was around the composition windows of the A360 alloys (Si:9–10 wt.%, Mg: 0.4–0.6 wt.%). The corresponding properties (UTS, YS, and EL) during the iteration processes were displayed in Supplementary Figure S4. The EI after the 5^th^ iteration was calculated and shown in Figure 6(f). The results show that the EI of models after the 5^th^ iteration reduced less than that from the last iteration, indicating that the uncertainty of the models is almost reaching a relative minimum. The maximum EI reduces from 24 of the 4^th^ iteration to 10 at the Al-10Si-0.5 Mg (optimal Sc 0.83 wt.%), which is located in the composition range of A360 alloy. Moreover, the predicted *Q* (374 ± 30 MPa) of the newly designed alloy (Al-10Si-0.5 Mg-0.83Sc) is very close to the *Q* of the existing A360–0.79Sc alloy (Al-9.5Si-0.5 Mg-0.79Sc, *Q* = 380 ± 30 MPa). It means that the prediction accuracy of the models is high, and the benefits of the next iterative optimization should be not large. Herein, the iterative optimization process can be stopped.

### 3.4. Discovery of hypoeutectic Al-xSi-yMg-zSc alloys with best mechanical properties

**Figure 7.**
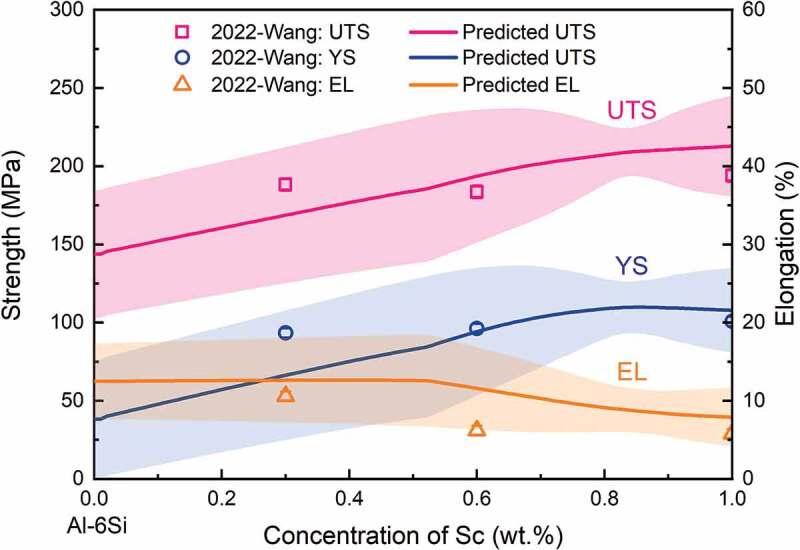
Validation of the iterated machine learning model results. The 5^th^ iterative model is used to predict the experimental values in Al-6si-*x*Sc alloys [25]. The error ranges of predicted results are plotted with 2 standard deviations of the mean value of 1000 ANN model results, corresponding to the confidence interval of 95%.

Moreover, considering that the experimental points are distributed in the commercial hypoeutectic Al-Si-Mg-Sc space (see Supplementary Figures S5 and S6), the trained ANN models should be applicable to predict the mechanical properties over the entire hypoeutectic Al-*x*Si-*y*Mg-*z*Sc composition space. In order to test the predictive ability of the current models at unknown points in the range of hypoeutectic Al-Si-Mg alloys, the lasted experimental points of Al-6Si-*x*Sc (*x* = 0.3, 0.6, 1 wt.%) alloys from the work of Wang et al [25]. were chosen as the predicted system. Figure 7 shows the predicted results based on the current models and experimental results for Al-6Si-*x*Sc alloys. The error ranges of predicted results are plotted with two standard deviations of the mean value of 1000 ANN model results, corresponding to the confidence interval of 95%. As shown in Figure 7, all of the experimental mechanical properties of Al-6Si-*x*Sc alloys are located within the 95% confidence interval of predictions. The results indicate that the current models also have a good predictive ability in the global composition space.

**Figure 8.**
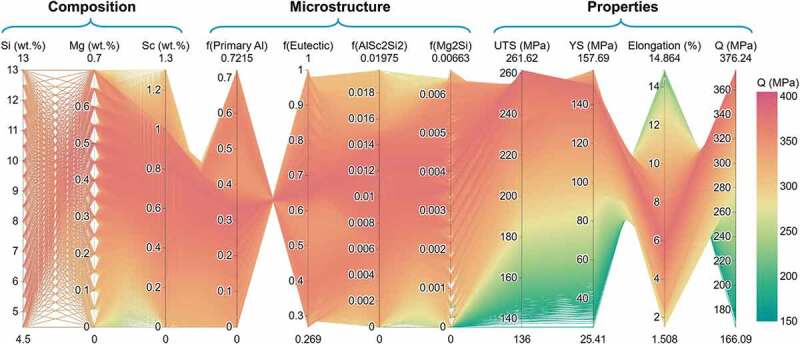
Quantitative relation ‘composition/process-microstructure-properties’ for hypoeutectic Al-Si-Mg-Sc casting alloys. The results were predicted by the 5^th^ iteration ANN models and plotted with a parallel diagram.

Therefore, the trained ANN models can be used to screen the optimal contents of Si, Mg, and Sc in the entire hypoeutectic Al-Si-Mg-Sc composition space. Figure 8 comprehensively presents the relation ‘composition/process-microstructure-properties’ for hypoeutectic Al-Si-Mg-Sc casting alloys using the 5^th^ iteration ANN models. Besides, the corresponding strengthening/toughening mechanisms in Al-Si-Mg-Sc alloys can be seen in Supplementary Figure S7, indicating that the Mg_2_Si phase, AlSc_2_Si_2_ phase, and eutectic (Al)+(Si) structure have a beneficial effect on the strength but a weak effect on the elongation, while the primary (Al) phase has the opposite effect.

**Figure 9.**
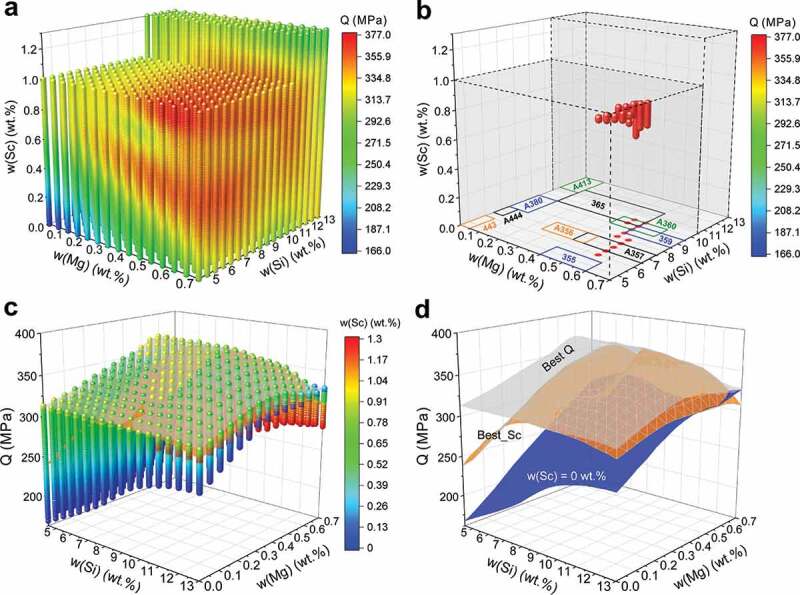
Discovery of hypoeutectic Al-Si-Mg-Sc casting alloys with the best comprehensive mechanical properties. (a) Predicted comprehensive mechanical property index *Q* of Al-Si-Mg-Sc alloys based on ANN models from the 5^th^ iteration; (b) Designed alloys with predicted comprehensive mechanical property index *Q* over 372 MPa based on ANN models from the 5^th^ iteration; (c) Predicted *Q* for hypoeutectic Al-Si-Mg alloys with the addition of Sc; (d) Comparison of predicted *Q* for hypoeutectic Al-Si-Mg alloys with and without optimal Sc.

The predicted comprehensive mechanical property index *Q* over the entire hypoeutectic Al-Si-Mg-Sc composition space was shown in Figure 9(a). The results demonstrate that the composition space for the alloys with the highest *Q* values locate around the range of 4.5–10 wt.% Si, 0.4–0.7 wt.% Mg and 0.2–1.0 wt.% Sc. Moreover, the alloys with the best comprehensive mechanical property can be screened over the entire hypoeutectic Al-Si-Mg-Sc composition space. Figure 9(b) presents the screened optimal contents of Si, Mg, and Sc in the entire hypoeutectic Al-Si-Mg-Sc composition space when the predicted *Q* is over 372 MPa, which is larger than 99.7% of the alloys. As can be seen from Figure 9(b), the composition space with maximum *Q* is mainly around the range of 6–10 wt.% Si, 0.5–0.6 wt.% Mg and 0.3–1.0 wt.% Sc, located in the composition space of the Sc-modified A357, 359, and A360 alloys. In addition, the predicted *Q* for hypoeutectic Al-Si-Mg alloys with the additional Sc is shown in Figure 9(c). In the figure, the color bar represents the content of Sc. The comparison of predicted *Q* for hypoeutectic Al-Si-Mg alloy without and with optimal Sc is shown in Figure 9(d). With the addition of optimal Sc based on the CT method, the comprehensive mechanical property of hypoeutectic Al-Si-Mg alloys is improved significantly, with *Q* values reaching maximum values in most regions. As can be seen in Figures 7 and 9(d), although there is relatively large uncertainty in the corner with minimum Mg and Si due to lack of data, the current models are adequate to design the alloys with desired high *Q*.

## 4. Conclusions

In this paper, we proposed a novel alloy design strategy integrating active learning with high-throughput CALPHAD calculations and key experiments to accurately explore the optimal contents of Si, Mg, and Sc in hypoeutectic Al-*x*Si-*y*Mg-*z*Sc casting alloys over high-dimensional composition space with the multi-objective optimization.

We first performed the high-throughput CALPHAD solidification simulations of ocean of hypoeutectic Al-Si-Mg-Sc casting alloys over a wide composition range to establish the quantitative relation ‘composition-process-microstructure’. Then, the relation ‘microstructure-mechanical properties’ of Al-Si-Mg-Sc hypoeutectic casting alloys was acquired using the active learning technique supported by key experiments designed by CALPHAD and Bayesian optimization samplings. Moreover, the uncertainties from data noise and models were quantitatively evaluated during modeling.

After a benchmark in A356-*x*Sc alloys, the results showed the microstructural features are better than composition features during modeling and Bayesian optimization samplings. Then, such a strategy was utilized to design the high-performance hypoeutectic Al-*x*Si-*y*Mg alloys with optimal Sc additions that were later experimentally validated. Finally, the present strategy was successfully extended to screen the optimal contents of Si, Mg, and Sc over high-dimensional hypoeutectic Al-*x*Si-*y*Mg-*z*Sc composition space, the composition space for maximum comprehensive mechanical property mainly locates in the range of 6–10 wt.% Si, 0.5–0.6 wt.% Mg and 0.3–1.0 wt.% Sc, conforming well with that of the Sc-modified A357, 359, and A360 alloys.

It is anticipated that the proposed strategy integrating active learning with high-throughput CALPHAD simulations and key experiments should be generally applicable to the efficient design of high-performance multi-component materials over high-dimensional composition space.

## Supplementary Material

Supplemental Material

## Data Availability

The data that support the findings of this study are available from the corresponding author upon reasonable request.

## References

[1] Davis JR. Aluminum and aluminum alloys. OH: ASM International; 1993.

[2] Kuchariková L, Tillová E, Chalupová M. The Si particles morphology in hypoeutectic Al-Si casts. Mater Today Proc. 2016;3(4):1031–17.

[3] Lathabai S, Lloyd PG. The effect of scandium on the microstructure, mechanical properties and weldability of a cast Al-Mg alloy. Acta Mater. 2002;50(17):4275–4292.

[4] Prukkanon W, Srisukhumbowornchai N, Limmaneevichitr C. Modification of hypoeutectic Al–Si alloys with scandium. J Alloys Compd. 2009;477(1–2):454–460.

[5] Prukkanon W, Srisukhumbowornchai N, Limmaneevichitr C. Influence of Sc modification on the fluidity of an A356 aluminum alloy. J Alloys Compd. 2009;487(1–2):453–457.

[6] Patakham U, Kajornchaiyakul J, Limmaneevichitr C. Grain refinement mechanism in an Al-Si-Mg alloy with scandium. J Alloys Compd. 2012;542:177–186.

[7] Patakham U, Kajornchaiyakul J, Limmaneevichitr C. Modification mechanism of eutectic silicon in Al-6si-0.3mg alloy with scandium. J Alloys Compd. 2013;575:273–284.

[8] Pandee P, Gourlay CM, Belyakov SA, et al. Eutectic morphology of Al-7si-0.3mg alloys with scandium additions. Metall Mater Trans A. 2014;45(10):4549–4560.

[9] Chanyathunyaroj K, Patakham U, Kou S, et al. Mechanical properties of squeeze-cast Al-7si-0.3mg alloys with Sc-modified Fe-rich intermetallic compounds. Rare Met. 2017;37(9):769–777.

[10] Chanyathunyaroj K, Patakham U, Kou S, et al. Microstructural evolution of iron-rich intermetallic compounds in scandium modified Al-7si-0.3mg alloys. J Alloys Compd. 2017;692:865–875.

[11] Lu Z, Zhang LJ. Thermodynamic description of the quaternary Al-Si-Mg-Sc system and its application to the design of novel Sc-additional A356 alloys. Mater Des. 2017;116:427–437.

[12] Chokemorh P, Pandee P, Limmaneevichitr C. Role of scandium additions in primary silicon refinement of hypereutectic Al–20Si alloys. Int J Cast Met Res. 2018;31(5):1–10.

[13] Pandee P, Gourlay CM, Belyakov SA, et al. AlSi_2_Sc_2_ intermetallic formation in Al-7Si-0.3Mg-xSc alloys and their effects on as-cast properties. J Alloys Compd. 2018;731:1159–1170.

[14] Puparattanapong K, Pandee P, Boontein S, et al. Fluidity and hot cracking susceptibility of A356 alloys with Sc additions. Trans Indian Inst Met. 2018;71(7):1583–1593.

[15] Lu Z, Zhang LJ, Wang J, et al. Understanding of strengthening and toughening mechanisms for Sc-modified Al-Si-(Mg) series casting alloys designed by computational thermodynamics. J Alloys Compd. 2019;805:415–425.

[16] Liu GC, Gao JB, Che C, et al. Optimization of casting means and heat treatment routines for improving mechanical and corrosion resistance properties of A356-0.54Sc casting alloy. Mater Today Commun. 2020;24:101227.

[17] Tsai YC, Chou CY, Lee SL, et al. Effect of trace La addition on the microstructures and mechanical properties of A356 (Al–7si–0.35mg) aluminum alloys. J Alloys Compd. 2009;487(1–2):157–162.

[18] Vončina M, Kores S, Mrvar P, et al. Effect of Ce on solidification and mechanical properties of A360 alloy. J Alloys Compd. 2011;509(27):7349–7355.

[19] Pandee P, Patakham U, Limmaneevichitr C. Microstructural evolution and mechanical properties of Al-7Si-0.3Mg alloys with erbium additions. J Alloys Compd. 2017;728:844–853.

[20] Knuutinen A, Nogita K, Mcdonald SD, et al. Modification of Al–Si alloys with Ba, Ca, Y and Yb. J Light Met. 2001;1(4):229–240.

[21] Riva S, Yusenko KV, Lavery NP, et al. The scandium effect in multicomponent alloys. Int Mater Rev. 2016;61(3):203–228.

[22] Kim M, Hong Y, Cho H. The effects of Sc on the microstructure and mechanical properties of hypo-eutectic Al−Si alloys. Met Mater Int. 2004;10(6):513–520.

[23] Kim M. Electron back scattering diffraction (EBSD) analysis of hypereutectic Al−Si alloys modified by Sr and Sc. Met Mater Int. 2007;13(2):103–107.

[24] Gaiselmann G, Stenzel O, Kruglova A, et al. Competitive stochastic growth model for the 3D morphology of eutectic Si in Al–Si alloys. Comput Mater Sci. 2013;69:289–298.

[25] Wang Z, Liu X, Zhu C, et al. Influence of the interaction between Si and Sc on the microstructure and tensile properties of as casted Al-Si-Sc alloys. J Alloys Compd. 2023;932:167650.

[26] Dorin T, Babaniaris S, Jiang L, et al. Stability and stoichiometry of L1_2_ Al_3_(Sc,Zr) dispersoids in Al-(Si)-Sc-Zr alloys. Acta Mater. 2021;216:117117.

[27] Wang R, Jiang S, Chen B, et al. Size effect in the Al_3_Sc dispersoid-mediated precipitation and mechanical/electrical properties of Al-Mg-Si-Sc alloys. J Mater Sci Technol. 2020;57:78–84.

[28] Zhang F, Qin A, Liu S, et al. Phase equilibria and solidification characteristics of the Al–Sc–Si alloys. J Mater Sci. 2015;51(3):1644–1658.

[29] Chen D, Xia C, Chen Z, et al. Thermodynamic, elastic and electronic properties of AlSc_2_Si_2_. Mater Lett. 2015;138:148–150.

[30] Muhammad A, Xu C, Xuejiao W, et al. High strength aluminum cast alloy: a Sc modification of a standard Al–Si–Mg cast alloy. Mater Sci Eng A. 2014;604:122–126.

[31] Pramod SL, Ravikirana, Prasada Rao AK, et al. Effect of Sc addition and T6 aging treatment on the microstructure modification and mechanical properties of A356 alloy. Mater Sci Eng A. 2016;674:438–450.

[32] Xu C, Xiao W, Hanada S, et al. The effect of scandium addition on microstructure and mechanical properties of Al–Si–Mg alloy: a multi-refinement modifier. Mater Charact. 2015;110:160–169.

[33] Yang S, Liu G, Zhong Y. Revisit the VEC criterion in high entropy alloys (HEAs) with high-throughput ab initio calculations: a case study with Al-Co-Cr-Fe-Ni system. J Alloys Compd. 2022;916:165477.

[34] Li J, Xie B, Fang Q, et al. High-throughput simulation combined machine learning search for optimum elemental composition in medium entropy alloy. J Mater Sci Technol. 2021;68:70–75.

[35] Liu F, Wang Z, Wang Z, et al. High-throughput method-accelerated design of Ni-based superalloys. Adv Funct Mater. 2022;32:2109367.

[36] Zhong J, Chen L, Zhang LJ. Automation of diffusion database development in multicomponent alloys from large number of experimental composition profiles. npj Comput Mater. 2021;7(1):35.

[37] Wei M, Tang Y, Zhang LJ, et al. Phase-field simulation of microstructure evolution in industrial A2214 alloy during solidification. Metall Mater Trans A. 2015;46(7):3182–3191.

[38] Park S, Kayani SH, Euh K, et al. High strength aluminum alloys design via explainable artificial intelligence. J Alloys Compd. 2022;903:163828.

[39] Yang C, Ren C, Jia Y, et al. A machine learning-based alloy design system to facilitate the rational design of high entropy alloys with enhanced hardness. Acta Mater. 2022;222:117431.

[40] Yi W, Liu GC, Lu Z, et al. Efficient alloy design of Sr-modified A356 alloys driven by computational thermodynamics and machine learning. J Mater Sci Technol. 2022;112:277–290.

[41] Yi W, Liu GC, Gao JB, et al. Boosting for concept design of casting aluminum alloys driven by combining computational thermodynamics and machine learning techniques. J Mater Inf. 2021;1(2):11.

[42] Zhang S, Yi W, Zhong J, et al. Computer alloy design of Ti modified Al-Si-Mg-Sr casting alloys for achieving simultaneous enhancement in strength and ductility. Materials. 2023;16(1):306. 10.3390/ma16010306 PMC9822033 36614645

[43] Gao JB, Zhong J, Liu GC, et al. A machine learning accelerated distributed task management system (Malac-Distmas) and its application in high-throughput CALPHAD computation aiming at efficient alloy design. Adv Powder Mater. 2022;1(1):100005.

[44] Liu P, Huang H, Antonov S, et al. Machine learning assisted design of γ′-strengthened Co-base superalloys with multi-performance optimization. npj Comput Mater. 2020;6(1):62.

[45] Chen Y, Tian Y, Zhou Y, et al. Machine learning assisted multi-objective optimization for materials processing parameters: a case study in Mg alloy. J Alloys Compd. 2020;844:156159.

[46] Dai R, Yang SL, Zhang TD, et al. High-throughput screening of optimal process parameters for PVD TiN coatings with best properties through a combination of 3-D quantitative phase-field simulation and hierarchical multi-objective optimization strategy. Front Mater. 2022;9:924294.

[47] Lookman T, Balachandran PV, Xue D, et al. Active learning in materials science with emphasis on adaptive sampling using uncertainties for targeted design. npj Comput Mater. 2019;5(1):21.

[48] Feng R, Zhang C, Gao MC, et al. High-throughput design of high-performance lightweight high-entropy alloys. Nat Commun. 2021;12(1):4329. 34267192 10.1038/s41467-021-24523-9 PMC8282813

[49] Andersson JO, Helander T, Höglund L, et al. Thermo-Calc & DICTRA, computational tools for materials science. Calphad. 2002;26(2):273–312.

[50] Fu H, Zhang H, Wang C, et al. Recent progress in the machine learning-assisted rational design of alloys. Int J Min Met Mater. 2022;29(4):635–644.

[51] Liu X, Xu P, Zhao J, et al. Material machine learning for alloys: applications, challenges and perspectives. J Alloys Compd. 2022;921:165984.

[52] Katsube R, Terayama K, Tamura R, et al. Experimental establishment of phase diagrams guided by uncertainty sampling: an application to the deposition of Zn–Sn–P films by molecular beam epitaxy. ACS Mater Lett. 2020;2(6):571–575.

[53] Liu Y, Wang L, Zhang H, et al. Accelerated development of high-strength magnesium alloys by machine learning. Metall Mater Trans A. 2021;52(3):943–954.

[54] Rao Z, Tung PY, Xie R, et al. Machine learning-enabled high-entropy alloy discovery. Science. 2022;378(6615):78–85. 36201584 10.1126/science.abo4940

[55] Amornsamankul S, Pimpunchat B, Triampo W, et al. A comparison of machine learning algorithms and their applications. Int J Simul Syst Sci Technol. 2019;20:8.

[56] Pedregosa F, Varoquaux G, Gramfort A, et al. Scikit-learn: machine learning in python. J Mach Learn Res. 2011;12:2825–2830.

[57] Paszke A, Gross S, Massa F, et al., editors. Pytorch: an imperative style, high-performance deep learning library. Advances in Neural Information Processing Systems. Vol. 32. Vancouver (BC): Curran Associates, Inc; 2019.

[58] Wen C, Zhang Y, Wang C, et al. Machine learning assisted design of high entropy alloys with desired property. Acta Mater. 2019;170:109–117.

[59] Xie J, Su Y, Xue D, et al. Machine learning for materials research and development. Acta Metall Sin. 2021;57(11):1343–1361.

[60] Gopakumar AM, Balachandran PV, Xue D, et al. Multi-objective optimization for materials discovery via adaptive design. Sci Rep. 2018;8(1):3738. 29487307 10.1038/s41598-018-21936-3 PMC5829239

[61] Solomou A, Zhao G, Boluki S, et al. Multi-objective Bayesian materials discovery: application on the discovery of precipitation strengthened NiTi shape memory alloys through micromechanical modeling. Mater Des. 2018;160:810–827.

[62] Greenhill S, Rana S, Gupta S, et al. Bayesian optimization for adaptive experimental design: a review. IEEE Access. 2020;8:13937–13948.

[63] Brochu E, Cora VM, De Freitas N. A tutorial on Bayesian optimization of expensive cost functions, with application to active user modeling and hierarchical reinforcement learning. 2010.

[64] Liu X, Zhang J, Pei Z. Machine learning for high-entropy alloys: progress, challenges and opportunities. Prog Mater Sci. 2023;131:101018.

[65] Xue D, Balachandran PV, Hogden J, et al. Accelerated search for materials with targeted properties by adaptive design. Nat Commun. 2016;7:11241. 27079901 10.1038/ncomms11241 PMC4835535

[66] Balachandran PV, Xue D, Theiler J, et al. Adaptive strategies for materials design using uncertainties. Sci Rep. 2016;6:19660. 26792532 10.1038/srep19660 PMC4726355

[67] Kaufman JG, Rooy EL. Aluminum alloy castings: properties, processes, and applications. Ohio (USA): ASM International; 2004.

[68] Schiessler EJ, Würger T, Lamaka SV, et al. Predicting the inhibition efficiencies of magnesium dissolution modulators using sparse machine learning models. Npj Comput Mater. 2021;7(1):193.

[69] Si S, Fan B, Liu X, et al. Study on strengthening effects of Zr-Ti-Nb-O alloys via high throughput powder metallurgy and data-driven machine learning. Mater Des. 2021;206:109777.

[70] Yuan R, Xue D, Xu Y, et al. Machine learning combined with feature engineering to search for BaTiO3 based ceramics with large piezoelectric constant. J Alloys Compd. 2022;908:164468.

[71] Li J, Zhang Y, Cao X, et al. Accelerated discovery of high-strength aluminum alloys by machine learning. Commun Mater. 2020;1(1):73.

[72] Drouzy M, Jacob S, Richard M. Interpretation of tensile results by means of quality index and probable yield strength. AFS Int Cast Metals J. 1980;5:43–50.

