# Supplementary material for: Accelerated discovery of high-performance Al-Si-Mg-Sc casting alloys by integrating active learning with high-throughput CALPHAD calculations
Source: Sci Technol Adv Mater. 2023 Apr 11;24(1):2196242. doi: 10.1080/14686996.2023.2196242 (PMC10101674; doi:10.1080/14686996.2023.2196242)
Supplement: Supplemental Material [file TSTA_A_2196242_SM2294.docx]

# Supplemental materials

## 1. Introduction

The relationship between the solidification paths of ternary Al-Si-Sc alloys and *w*(Si)/*w*(Sc) are shown in **Fig.S1** and **Table S1**.


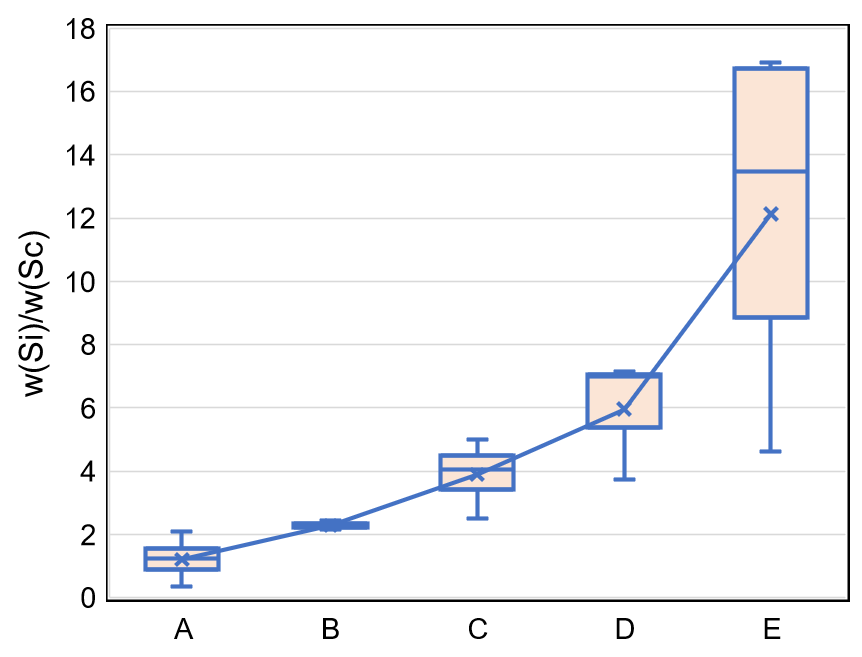


**Figure S1.** Relationship between the solidification paths of ternary Al-Si-Sc alloys and *w*(Si)/*w*(Sc) [[1](#_ENREF_1)], while the A-E represent the solidification pathways shown in **Table S1**.

**Table S1** Detected primary phase and solidification paths of ternary Al-Si-Sc alloys [[1](#_ENREF_1)]

| Type | Primary phase | Solidification pathways |
| --- | --- | --- |
| A | Al_3_Sc | L → Al_3_Sc, L → (Al) + Al_3_Sc, L + Al_3_Sc → (Al) + τ,  L → (Al) + τ, L → (Al) + τ + (Si) |
| B | Al_3_Sc | L → Al_3_Sc, L + Al_3_Sc → τ, L + Al_3_Sc → (Al) + τ,  L → (Al) + τ, L → (Al) + τ + (Si) |
| C | τ (AlSc_2_Si_2_) | L → τ, L → (Al) + τ, L → (Al) + τ + (Si) |
| D | (Al) | L → (Al), L → (Al) + τ, L → (Al) + τ + (Si) |
| E | (Al) | L → (Al), L → (Al) + (Si), L → (Al) + τ + (Si) |

The contents of Si and Mg of ten typical commercial hypoeutectic Al-Si-Mg casting alloys referred to in this study are shown in **Table S2**.

**Table S2** Contents of Si and Mg of ten typical commercial hypoeutectic Al-Si-Mg casting alloys [[2](#_ENREF_2)].

| Commercial alloys | Si (wt.%) | Mg (wt.%) |
| --- | --- | --- |
| 355 | 4.5―5.5 | 0.40―0.60 |
| A356 | 6.5―7.5 | 0.25―0.45 |
| A357 | 6.5―7.5 | 0.45―0.60 |
| 359 | 8.5―9.5 | 0.50―0.70 |
| A360 | 9.0―10.0 | 0.40―0.60 |
| 365 | 9.5―11.5 | 0.10―0.50 |
| A380 | 7.5―9.5 | 0―0.10 |
| A413 | 11.0―13.0 | 0―0.10 |
| 443 | 4.5―6.0 | 0―0.05 |
| A444 | 6.5―7.5 | 0―0.05 |

## 2. Methods

### 2.1 High-throughput CALPHAD calculations

By coupling Malac-Distmas with CALPHAD software, such as Thermo-Calc [[3](#_ENREF_3)] and Pandat [[4](#_ENREF_4)], the HTCs of thermodynamic calculations, such as Gibbs free energy, phase diagram, Scheil-Gulliver simulation, property diagram, thermophysical properties, diffusion simulation, and precipitation simulation, and so on, can be **automatically** performed in a user-defined compositional space, and all the calculated/simulated results will be stored in an SQLite format database. Moreover, the machine learning can be also embedded in Malac-Distmas to densify the output data, thus reducing the calculation/simulation amount and accelerating high-throughput calculations.

### 2.2 Machine learning technique

RF and ANN are the most common approaches in machine learning. RF is an ensemble learning method that merges tree predictors. It is used not only to fast construct the relationship between inputs and outputs, but also to analyze the feature importance of the inputs, which is beneficial for the feature selection. However, RF is not suitable for high dimensional sparse features and big data. ANN is similar to the black box which is related to an output set with an input set through artificial neural units. Although it is difficult to interpret the output results from ANN, ANN is able to approximate the arbitrarily complex nonlinear function and has better performance with big data.

The basic strategy of machine learning-assisted material design consists of data collection, feature engineering, model construction and validation, and material design. For feature engineering, Pearson correlation coefficient, features importance from a tree-based machine learning model, and domain knowledge are used to select features.

Pearson correlation coefficient, *p*, can be used to evaluate the correlation of features:

$p=\frac{\sum_{i=1}^{n} (x_{i}-\bar{x})(y_{i}-\bar{y})}{\sqrt{\sum_{i=1}^{n} (x_{i}-\bar{x})^{2}}\sqrt{\sum_{i=1}^{n} (y_{i}-\bar{y})^{2}}}$ (S1)

Here, *n* is the sample size, *x_i_* and *y_i_* are the individual sample points indexed with *i*, $\bar{x}$ and $\bar{y}$ are the sample mean. The *p*-value varies from -1 to 1. The higher the absolute value of *p*, the greater the correlation between the two features.

## 3. Results and Discussion

### 3.1 HTC results of Scheil solidification

Moreover, based on the HTC results, the optimal Sc content for each Al-Si-Mg hypoeutectic casting alloy can be designed with the same criterion and plotted as a plane. In order to conveniently use the optimal Sc content plane shown in **Figure 2** of manuscript, the nonlinear surface fitting was applied to fit the calculated results for the optimal additive amount of Sc in the commercial hypoeutectic Al-Si-Mg casting alloys. And the surface fitting results were as followed:

*w*(Sc)_opt_ = 0.09989×*w*(Si) – 0.16948×*w*(Mg) – 0.07436 (1)

where *w*(Sc)_opt_ is the optimal Sc content for each hypoeutectic Al-Si-Mg casting alloy, *w*(Si) and *w*(Mg) are the Si and Mg content (wt.%). The adjusted coefficient square (R^2^) values are 0.99884 and the reduced Chi-sqr values are 7.9993×10^-5^, the fitting results are in great agreement with the origin results. Based on the HTC results for Sc-additional commercial hypoeutectic Al-Si-Mg casting alloy shown in **Figure 2** of manuscript, the optimal Sc content for a series of commercial hypoeutectic Al-Si-Mg casting alloy can be used to guide the optimal mechanical properties in experiments. For instance, Kim *et al.* [[5](#_ENREF_5)] experimental results showed that the hardness reached the maximum value when Sc content equals 0.8 wt.% in Al-8.5Si casting alloy, in which Sc contents were 0.0, 0.2, 0.4, 0.8, and 1.6 wt.% in experiments. The experimental optimal addition amount of Sc is very close to the calculated optimal Sc content of 0.772 wt.% for Al-8.5Si alloy. Therefore, the HTC results for optimal Sc-additional in commercial hypoeutectic Al-Si-Mg are efficient and useful for guiding the design of Sc-additional Al-Si-Mg alloy composition.

### 3.2 Discovery of series of hypoeutectic Al-Si-Mg-*x*Sc alloys


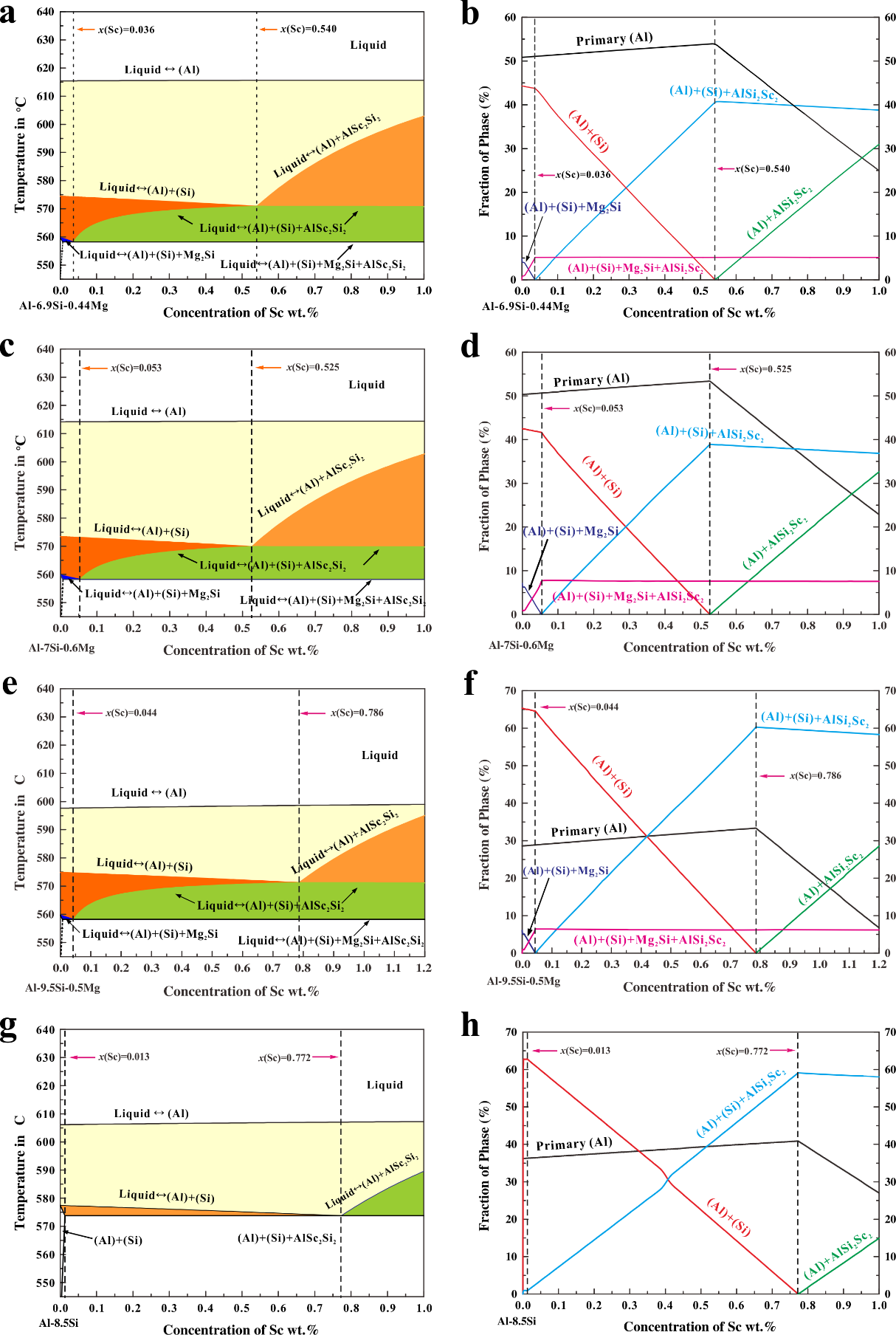


**Figure S2.** Solidification diagram and phase fraction diagram of commercial hypoeutectic casting Al-Si-Mg alloys with the addition of Sc: (a, b) A356, (c, d) A357, (e, f) A360, (g, h) A380.


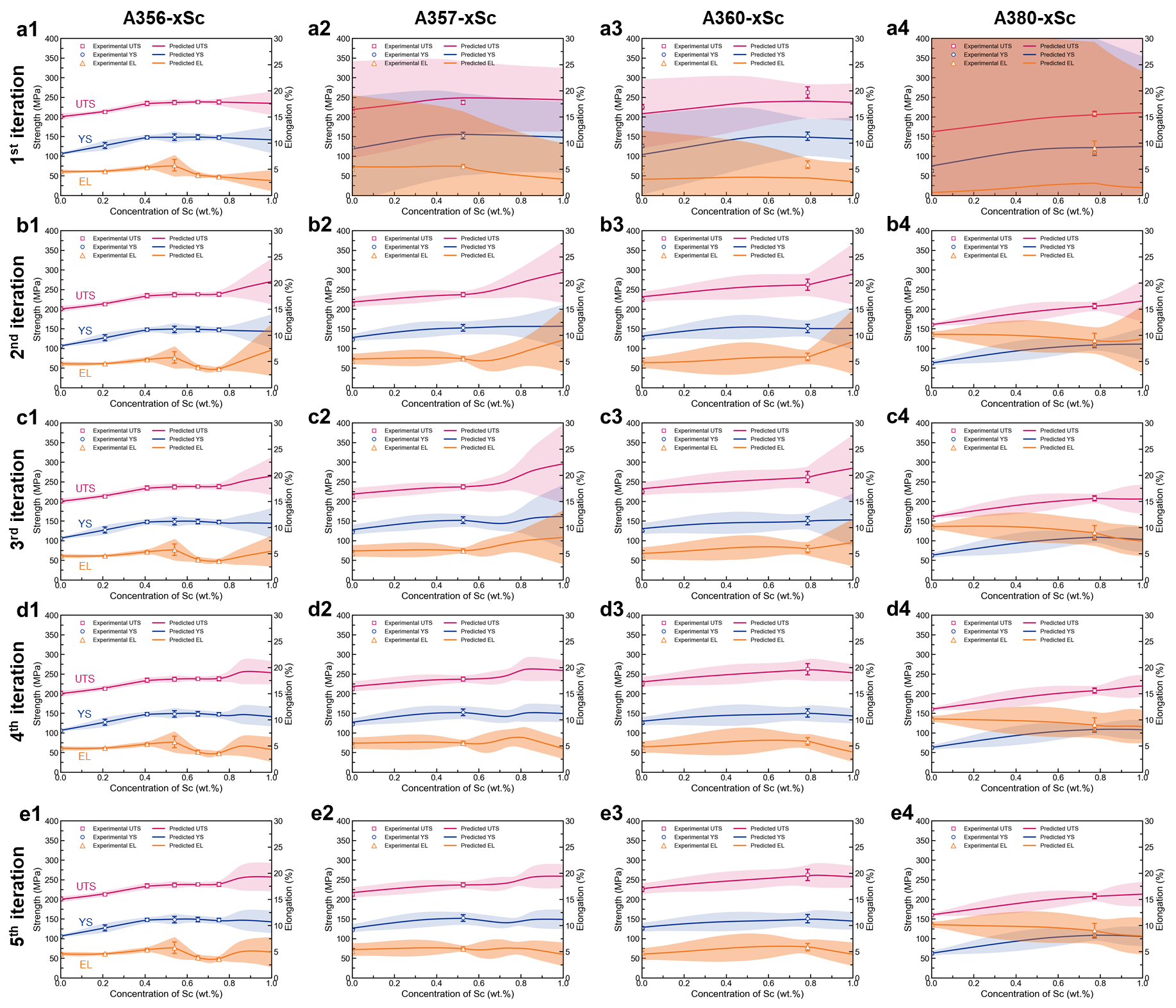


**Figure** **S3.** Predicted properties of series of Sc-modification Al-Si-Mg casting alloys (A356-*x*Sc, A357-*x*Sc, A360-*x*Sc, A380-*x*Sc) based on 1000 ANN models with microstructure feature group and considering data noise during the active learning processing: (a) Iteration 1, (b) Iteration 2, (c) Iteration 3, (d) Iteration 4, (e) Iteration 5.

### 3.3 Discovery of hypoeutectic Al-*x*Si-*y*Mg-*optimal* Sc alloys

**Figure S4** shows the predicted results of ANN models and experimental mechanical properties for the hypoeutectic Al-Si-Mg alloys with optimal Sc content during active learning processes, including 3^rd^ iteration, 4^th^ iteration, and 5^th^ iteration. The gray surfaces represent the 95% confidence interval of the predictions. As can be seen from **Figure S4a**, after the 3^rd^ iteration, the predictions of ANN models are surfaces related to the composition and can describe the existing experimental data well in terms of UTS, YS, and EL. The predictions from 3^rd^ iteration show that the compositions with maximum UTS and YS are located in the corner with maximum Si and Mg content, while the composition with maximum EL is located in the corner with minimum Si and Mg, and there is a large uncertainty in both corners. Then, Bayesian optimization sampling, which allows to balance the exploration (maximum Q) and exploitation (maximum uncertainty), was used to design new experimental data to improve the models. A1 alloy was designed based on the results of 3^rd^ iteration and fed back the dataset for the 4^th^ iteration, as shown in **Figure S4b**. A2 alloys were designed based on the results of 4^th^ iteration and fed back the dataset for the 5^th^ iteration, as shown in **Figure S4c**. During the iteration processes, the accuracy and uncertainty of prediction were continuously improved. After the 5^th^ iteration, the predictions of models stabilized when considering a maximum comprehensive mechanical property, although there is a large uncertainty in the corner of minimum Si and Mg content.


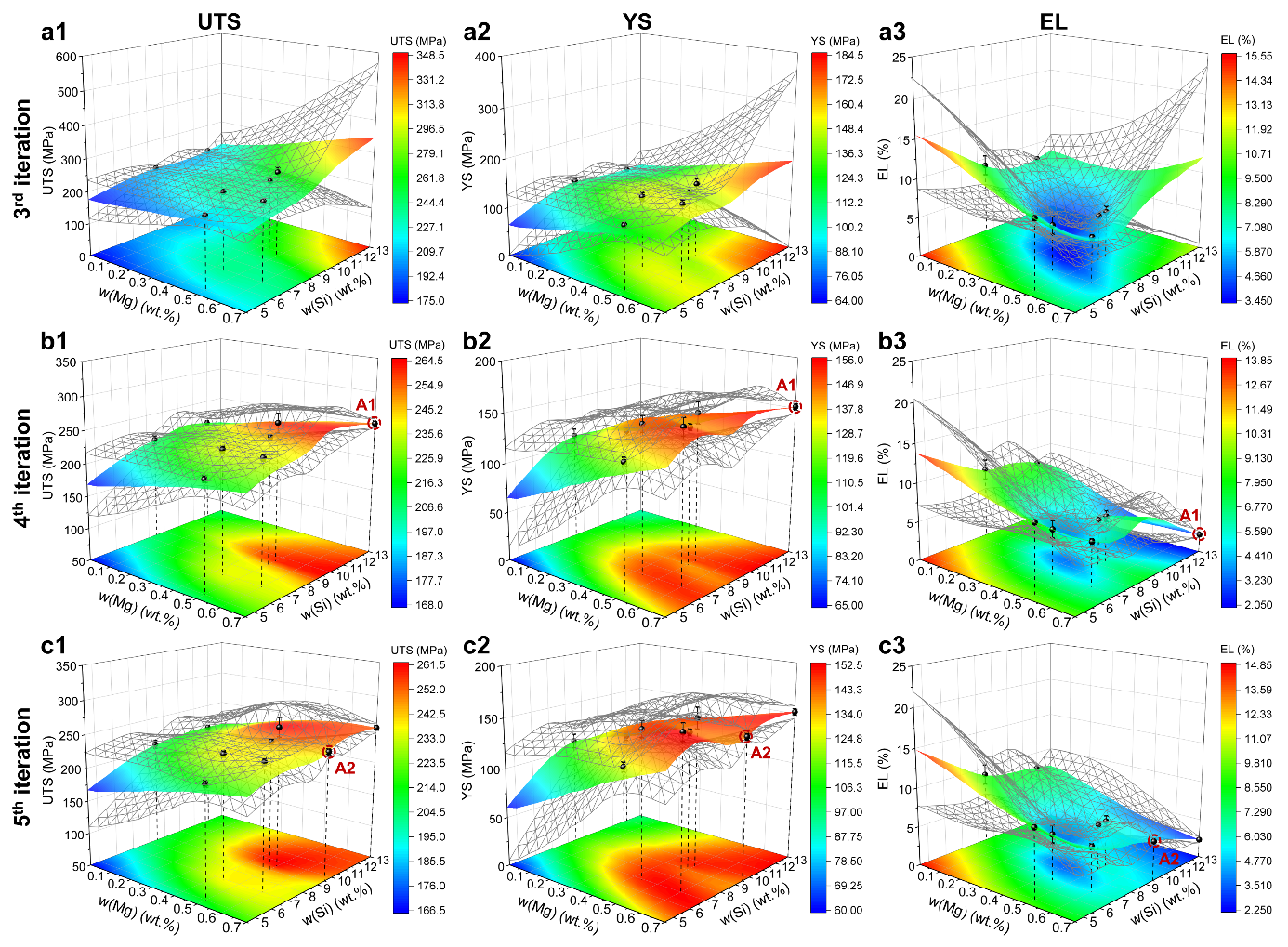


**Figure S4.** Predicted results of ANN models and experimental mechanical properties for the hypoeutectic Al-Si-Mg alloys with optimal Sc content during active learning processes: (a) 3^rd^ iteration, (b) 4^th^ iteration, (c) 5^th^ iteration. The gray surfaces represent the 95% confidence interval of the predictions.

### 3.4 Discovery of hypoeutectic Al-*x*Si-*y*Mg-*z*Sc alloys


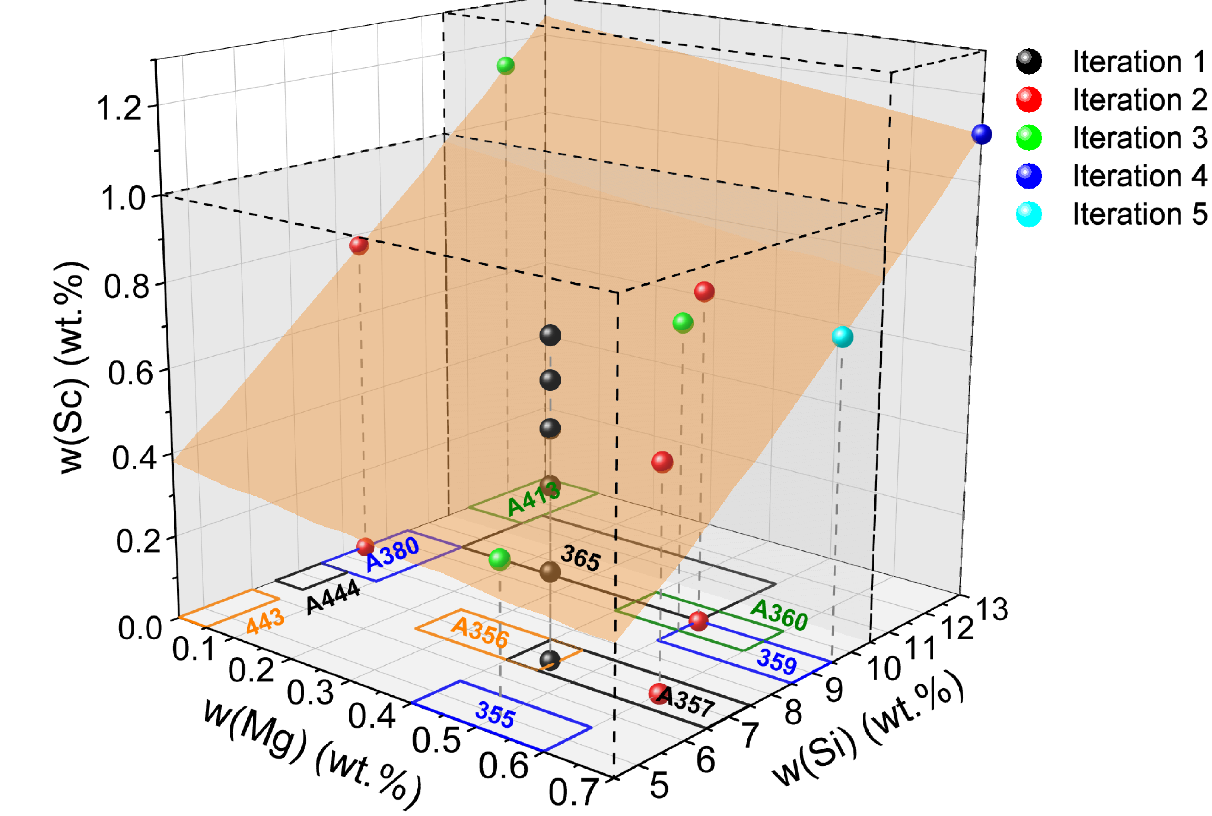


**Figure S5.** Experimental composition points of Sc-modified hypoeutectic Al-Si-Mg alloys during the active learning process.


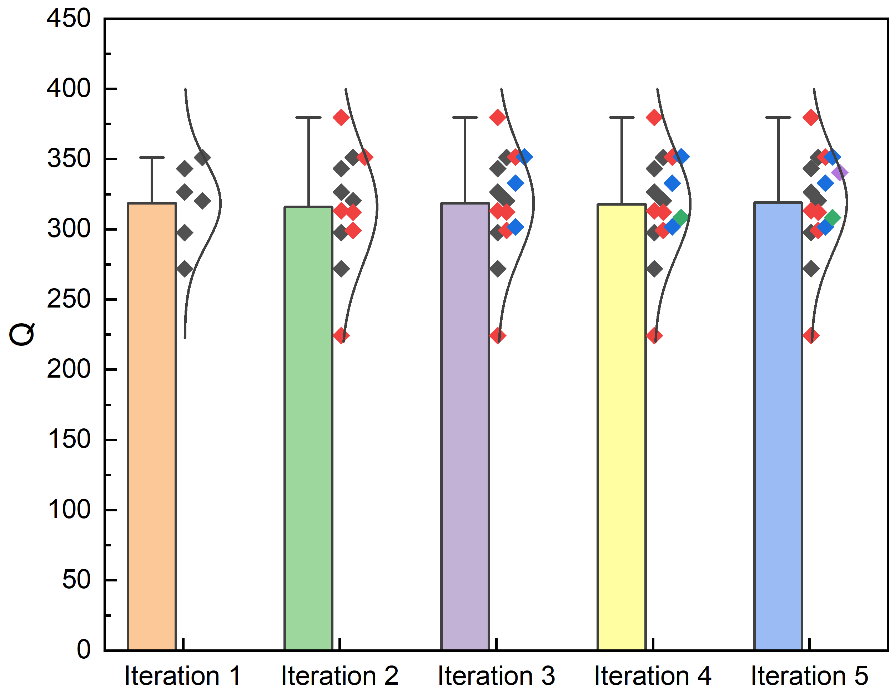


**Figure S6.** Distribution of comprehensive mechanical property Q for Sc-modified hypoeutectic Al-Si-Mg alloys during the active learning process.

### 3.5 Further understanding of strengthening/toughening mechanisms in hypoeutectic Al-Si-Mg-Sc alloys

UTS: *k* _Mg2Si_ > *k* _AlSc2Si2_ > *k* _Eutectic (Al+Si)_ > 0 > *k* _Primary (Al)_

YS: *k* _Mg2Si_ > *k* _AlSc2Si2_ > *k* _Eutectic (Al+Si)_ > 0 > *k* _Primary (Al)_

EL: *k* _Primary (Al)_ > 0 > *k* _Eutectic (Al+Si)_ > *k* _AlSc2Si2_ > *k* _Mg2Si_


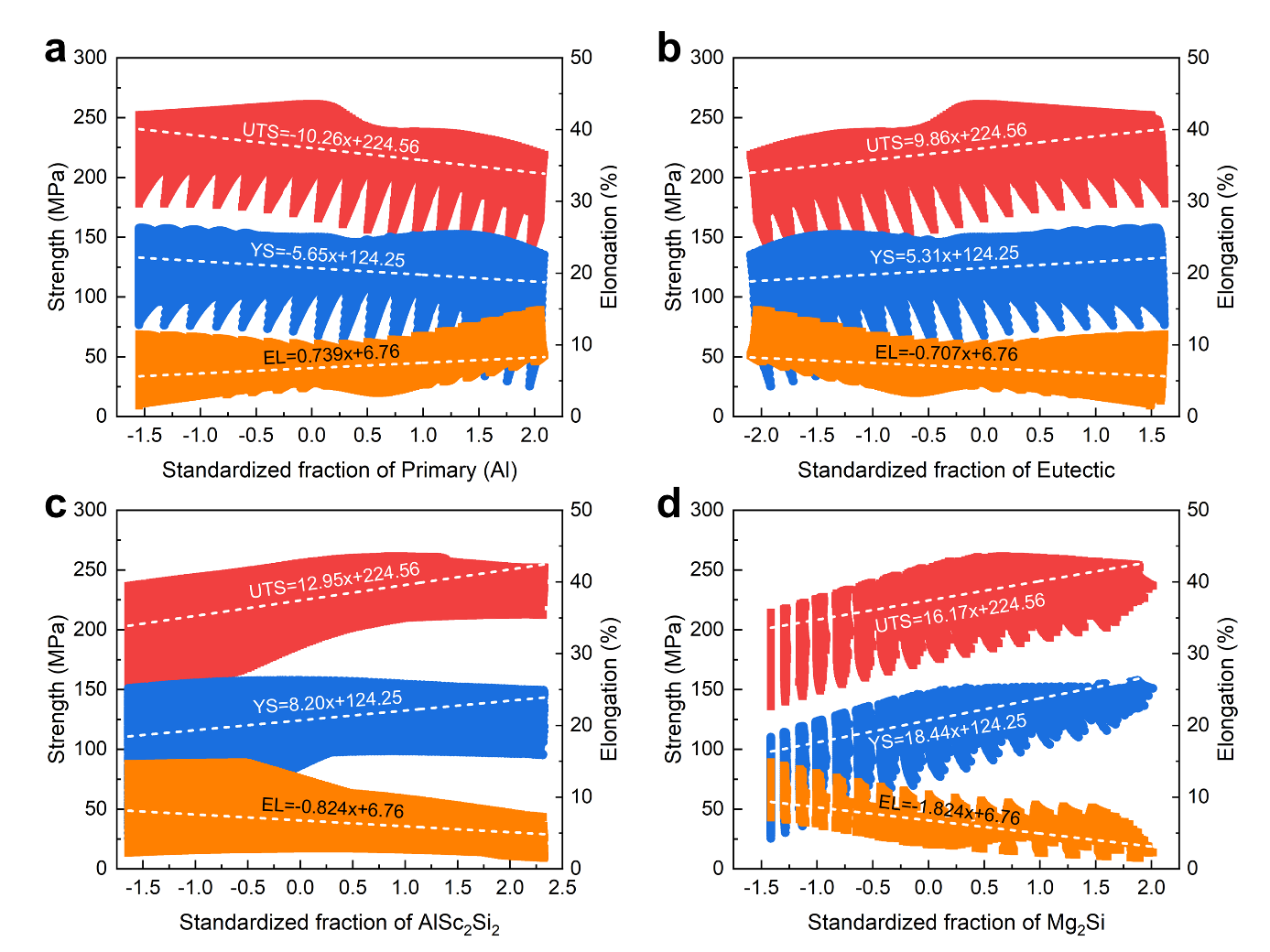


**Figure S7.** Relations between different mechanical properties and volume fractions of different phases/structures after standardization. (a) primary (Al); (b) Eutectic (Al)+(Si); (c) AlSc_2_Si_2_; (d) Mg_2_Si.


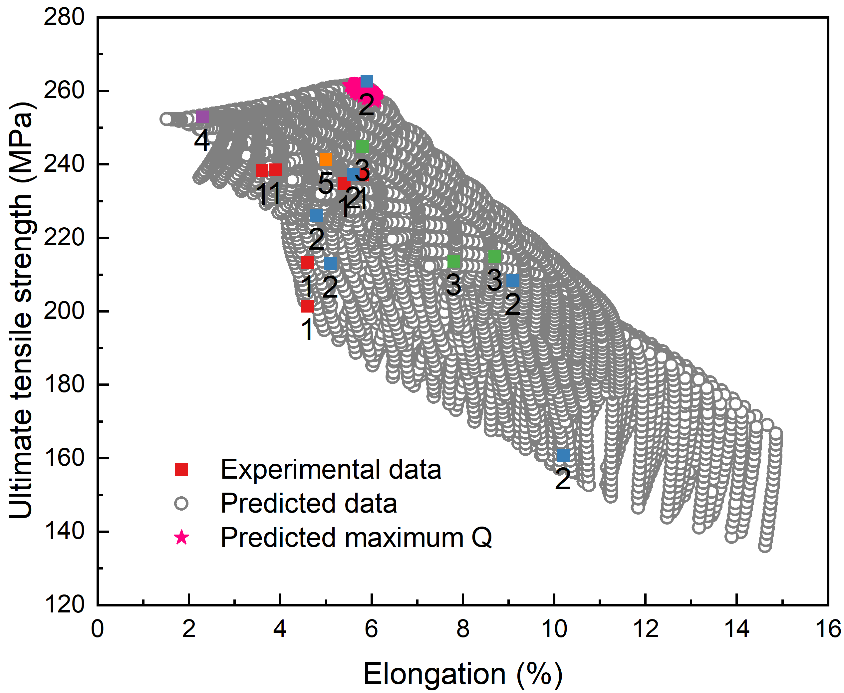


**Figure S8.** Summary of UTS and elongation to fracture for experimental data and predicted data of Sc-modified hypoeutectic Al-Si-Mg alloys.

## References

1. Zhang F, Qin A, Liu S, et al. Phase equilibria and solidification characteristics of the Al–Sc–Si alloys. J Mater Sci. 2015;51(3):1644-1658.

2. Kaufman JG, Rooy EL. Aluminum alloy castings: properties, processes, and applications. Ohio, USA: ASM International; 2004.

3. Thermo Calc Software. Available from: <http://www.thermocalc.com>

4. Cao W, Chen SL, Zhang F, et al. PANDAT software with PanEngine, PanOptimizer and PanPrecipitation for multi-component phase diagram calculation and materials property simulation. Calphad. 2009;33(2):328-342.

5. Kim M, Hong Y, Cho H. The effects of Sc on the microstructure and mechanical properties of hypo-eutectic Al−Si alloys. Met Mater Int. 2004;10(6):513-520.
